# Heterogeneous pathway activation and drug response modelled in colorectal-tumor-derived 3D cultures

**DOI:** 10.1371/journal.pgen.1008076

**Published:** 2019-03-29

**Authors:** Dirk Schumacher, Geoffroy Andrieux, Karsten Boehnke, Marlen Keil, Alessandra Silvestri, Maxine Silvestrov, Ulrich Keilholz, Johannes Haybaeck, Gerrit Erdmann, Christoph Sachse, Markus Templin, Jens Hoffmann, Melanie Boerries, Reinhold Schäfer, Christian R. A. Regenbrecht

**Affiliations:** 1 Laboratory of Molecular Tumor Pathology, Institute of Pathology, Charité Universitätsmedizin Berlin, Berlin, Germany; 2 German Cancer Consortium (DKTK), German Cancer Research Center (DKFZ), Heidelberg, Germany; 3 Institute of Molecular Medicine and Cell Research, Albert-Ludwigs-University Freiburg, Freiburg, Germany; 4 Eli Lilly and Company, Lilly Research Laboratories, Oncology Translational Research, New York, NY, United States of America; 5 EPO Experimental Pharmacology and Oncology Berlin-Buch GmbH, Berlin, Germany; 6 cpo—Cellular Phenomics & Oncology Berlin-Buch GmbH, Berlin, Germany; 7 Charité Comprehensive Cancer Center, Berlin, Germany; 8 Department of Pathology, Otto-von-Guericke University Magdeburg, Magdeburg, Germany; 9 Department of Pathology, Neuropathology, and Molecular Pathology, Medical University of Innsbruck, Austria; 10 Diagnostic & Research Center for Molecular BioMedicine, Institute of Pathology, Medical University of Graz, Austria; 11 NMI TT Pharmaservices, Berlin, Germany; 12 ASC Oncology GmbH, Berlin, Germany; 13 NMI Natural and Medical Sciences Institute at the University of Tübingen, Reutlingen, Germany; Institute of Cancer Research, UNITED KINGDOM

## Abstract

Organoid cultures derived from colorectal cancer (CRC) samples are increasingly used as preclinical models for studying tumor biology and the effects of targeted therapies under conditions capturing *in vitro* the genetic make-up of heterogeneous and even individual neoplasms. While 3D cultures are initiated from surgical specimens comprising multiple cell populations, the impact of tumor heterogeneity on drug effects in organoid cultures has not been addressed systematically. Here we have used a cohort of well-characterized CRC organoids to study the influence of tumor heterogeneity on the activity of the KRAS/MAPK-signaling pathway and the consequences of treatment by inhibitors targeting EGFR and downstream effectors. MAPK signaling, analyzed by targeted proteomics, shows unexpected heterogeneity irrespective of *RAS* mutations and is associated with variable responses to EGFR inhibition. In addition, we obtained evidence for intratumoral heterogeneity in drug response among parallel “sibling” 3D cultures established from a single *KRAS*-mutant CRC. Our results imply that separate testing of drug effects in multiple subpopulations may help to elucidate molecular correlates of tumor heterogeneity and to improve therapy response prediction in patients.

## Author summary

Commonly occurring genetic alterations and patient-specific genetic features are increasingly used to predict the possible action of targeted cancer therapies. Although several lines of evidence have suggested that preclinical and clinical responses concur, the heterogeneity of tumors remains a severe obstacle in routinely translating preclinical data to patient treatments. Here we present a rapid work flow that integrates drug testing of three-dimensional patient tumor-derived (organoid) cultures and assessment of their genetic make-up as well as that of their donor tumors by amplicon sequencing and targeted proteomics. While the organoid cultures largely recapitulated the genomic profiles of donor tumors, the overall treatment responses and inhibitor effects on the intracellular signaling system were quite variable. Notably, organoid cultures obtained by synchronous multi-regional sampling of the same colorectal tumor showed an up to 30-fold difference in drug response. A combinatorial drug treatment improved the response. These data were confirmed in matched mouse xenograft models from the same tumor. Our findings may help to refine preclinical testing of individual tumors by modelling heterogeneity in cultures, to better understand therapeutic failure in clinical settings and to find ways to overcome treatment resistance.

## Introduction

Colorectal cancer (CRC) is the third most common cancer worldwide. The major molecular alterations driving cancer of the colonic epithelium involve dysfunction of the WNT/APC and KRAS/MAPK pathways, DNA repair and methylation, and chromosomal instability [1, 2]. Concerted analysis of colorectal cancer transcriptomes has identified consensus molecular subtypes (CMS) that comprise genes controlling cell-type-specific functions, signaling pathways, and, in part, prognosis-relevant characteristics [3, 4]. CMS1 features genes involved in hyper-mutation and hyper-methylation. CMS2 represents the properties of epithelial tumor characteristics mainly driven by WNT/MYC signaling. CMS3 characterizes *KRAS*-mutated CRC and reflects genes controlling metabolic pathways. CMS4 exhibits expression of stromal components and activation of TGF-beta and VEGFR pathways. Like in other cancer entities, the administration of targeted drugs to colorectal cancer patients is based on the molecular profile of their tumors. Mutations in *RAS* gene family members or *BRAF* turn out to be negative predictors for anti-receptor tyrosine kinase therapies [5], while at least a subset of *RAS* wild-type tumors shows a therapeutic response [6].

Three-dimensional cell culture systems provide accurate and physiologically relevant models for studying the biology of diseases, and they support clinical research as well as drug development [7]. Recently, several groups have described patient-derived colorectal cancer organoids as a discovery platform for therapeutics and for validating the predicted impact of molecular features on therapy responses. Since the tissue architecture, tumor cell-specific genomic alterations, and consensus molecular signatures are essentially maintained in organoid cultures, these models are an excellent source for studying tumor biology in general under conditions reflecting clinically manifested heterogeneities of mutational patterns and epigenetic alterations [8–12]. For example, we identified the hedgehog pathway as a critical driver of colon cancer stem cell survival and tumorigenesis [13]. In scenarios approximating clinical behavior, drug treatments of CRC organoid cultures can even be used to predict personalized therapeutic options for individual patients [10]. A recent report has indeed documented the close relationship between drug effects observed in patient-tumor-specific organoid cultures and clinical responses in donor patients enrolled in clinical trials [14]. CRC organoid cultures also recapitulated the clinically well-known resistance of *KRAS*-mutant cells toward anti-receptor kinase therapies and showed limited effects of combinatorial targeted therapies against KRAS pathway effectors [15].

It is well known that the molecular diagnosis of primary and metastatic tumors, typically based on single biopsies representing snapshots of ongoing tumor evolution, may be compromised by intratumor heterogeneity (ITH) [16, 17]. Multi-region sampling of solid tumors revealed substantial ITH, as indicated by diverse patterns of cancer gene mutations [18] and copy number alterations [19] in different tumor areas. Individual tumors may harbor up to 10 or more preexisting subpopulations. Tumor evolution is assumed to be based on multiple initial alterations [20], and spatially separated subpopulations of cancer cells are generated during tumor progression [21]. Therefore, ITH is very likely to significantly influence initiation, establishment, fitness, and molecular characteristics of patient-tumor-derived organoid cultures.

Here, we used a previously characterized set of CRC organoids [11], supplemented by novel models, to investigate the impact of intertumor and intratumor heterogeneity on drug response. We chose to analyze the mutation status of the organoids by targeted amplicon sequencing, because interrogating a limited number of driver genes is often used as the standard diagnostic procedure [22, 23]. In addition, we analyzed signaling pathways in a subset of organoid models by targeted proteomics [24]. We correlated mutation patterns and pathway activity with the response to selected compounds targeting receptor tyrosine kinases, the RAS/MAPK, and PIK3CA pathway. Finally, we modelled the consequences of ITH on tumor cell growth and drug response in independently generated “sibling” cultures from the same donor tumor *in vitro* and *in vivo*.

## Results

### Establishment and molecular characterization of patient CRC-derived organoid cultures

**Fig 1:**
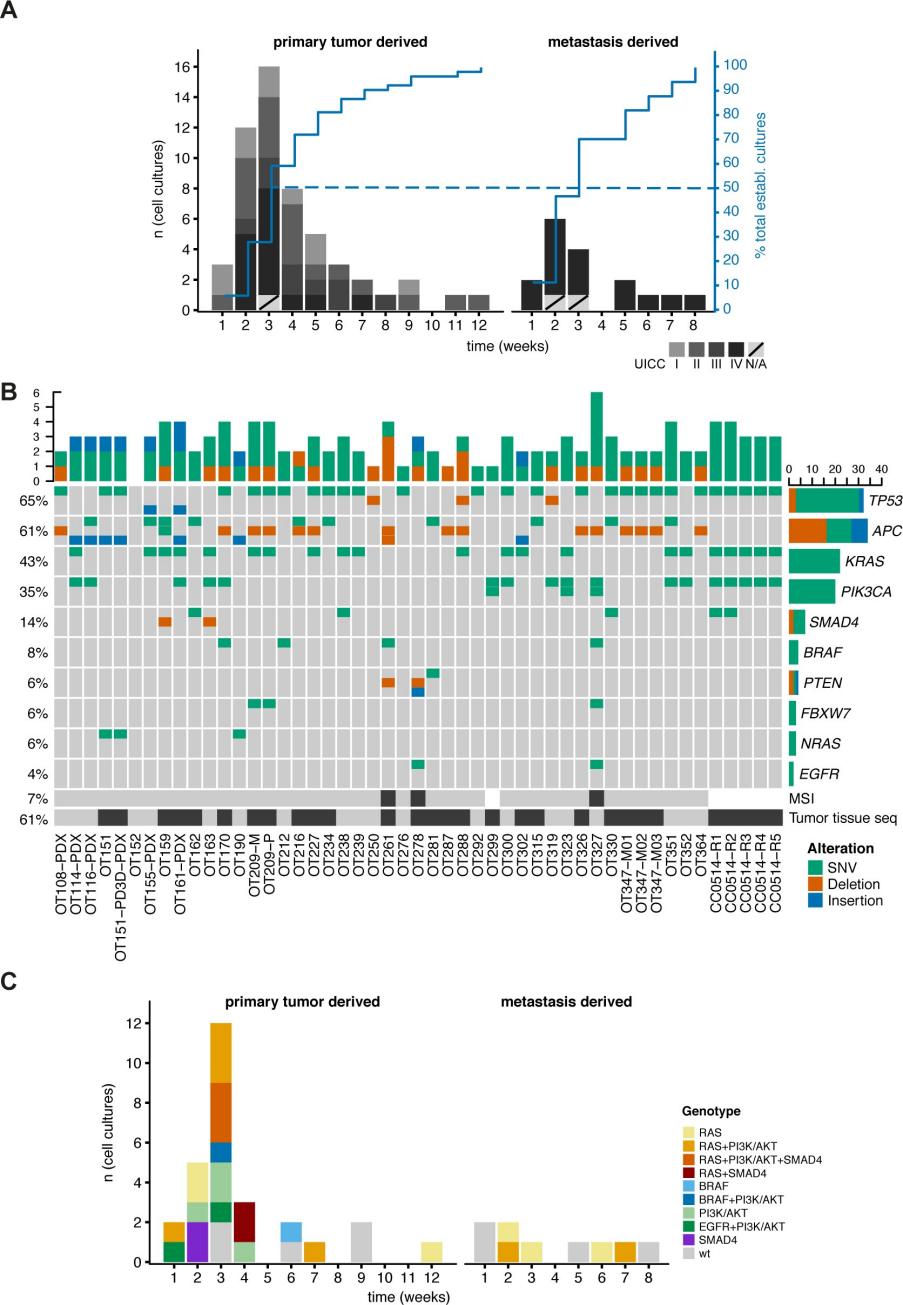
PD3D culture generation statistics and genomic analysis. (**A**) Duration (weeks) from culture initiation to first passage (primary tumors n = 54; metastatic tumors n = 17). UICC stages are indicated in the grey scale; metastases are generally considered as stage IV. (**B**) Mutation of key genes affected in CRC found via cancer gene panel sequencing in 49 patient-derived three-dimensional (PD3D) cultures and 29 matched original tumor samples (indicated with a black box at the bottom). The percentage of organoid cultures harboring a mutation in a given gene is shown on the left side, the overall number of mutations per gene is given on the right as a bar chart. Microsatellite instability was found in 3 of 43 tested samples (7%), indicated by a black box below the matrix. (**C**) For sequenced organoid cultures, duration of culturing until the first passages correlated with critical mutations. Color code is given in the figure (wt = no mutations in MAPK/PI3K-AKT/TGFβ-SMAD4 pathways).

Currently, our collection of patient-tumor-derived cell culture models comprises 91 organoids originating from 158 primary or metastatic tumor specimens (**S1 Table**). Due to the limitations of clinical material, not all tumor biopsies obtained gave rise to proliferation-competent organoid cultures. The time intervals required for the first transfer of primary to secondary cultures varied substantially but was not related to the UICC stage of donor tumors (**Fig 1A**, **S1 Fig**). Sufficient cell material for subsequent assays was available in 70% (51/71) of cultures 3 to 4 weeks post-explantation, while the remaining cultures required 5 to 12 weeks. Successfully established cultures maintained typical CRC marker expression and an adenoma-like architecture that retains higher-order organization and apical-basal polarity of the colonic epithelium, as reported earlier [11]. In view of the reported genomic heterogeneity of CRCs, the cultures were expected to be invariably polyclonal during establishment and early passaging.

We evaluated 50 cancer genes by ultra-deep targeted amplicon sequencing [25] in 49 organoids and 29 matched donor tumors (**Fig 1B**, **S2 Table**). Donor tumors and derived models both harbored common driver mutations in EGFR/RAS/RAF/MEK, PIK3CA/AKT, and TGF-beta signaling pathways, confirming previous exome sequencing results [11]. There were few exceptions, most likely due to population dynamics in the original tumor tissues [11, 18, 19]. Donor tumor tissue OT288 harbored an *ABL1* mutation at a frequency of 6.3%, not detected in the corresponding organoid culture. The *SMAD4* and *PIK3CA* mutations manifested in OT159 and OT161 cultures were not detected in the donor materials. A low-frequency *APC* mutation in tumor OT281, represented by only 3% of sequencing reads, was retrieved as a homozygous mutation in the corresponding organoid culture. As the received samples represented a macro-dissected admixture of tumor cells and adjacent healthy cells of different cell types, increased mutation frequencies *in vitro* were most likely due to enrichment of tumor cells by the used culture system and medium.

Notably, we observed a trend for earlier passaging in cultures that had accumulated mutations in *RAS*, *PI3K/AKT*, or *SMAD4*, as indicated by robust establishment and short culture intervals (**Fig 1C**).

### *In vitro* drug response of organoid cultures is heterogeneous and independent of their *KRAS* oncogene status and pathway activation

**Fig 2:**
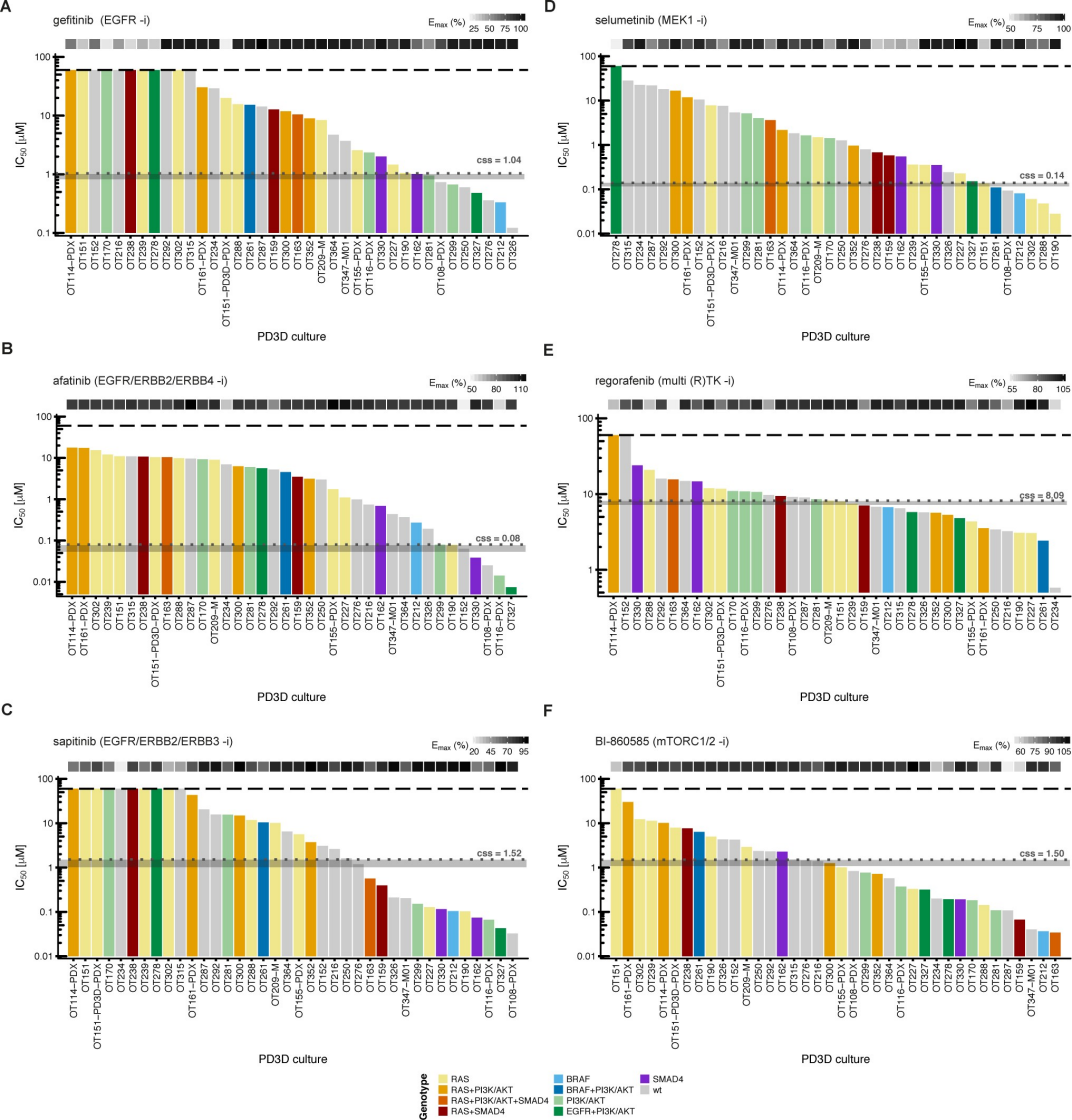
*In vitro* drug response waterfall plots of 38 organoid cultures show individual patterns of resistance and sensitivity. **IC**_**50**_ **values for 38 *in vitro* models were determined with a semi-automated drug response assay platform. The lower and upper assay cutoffs were 0.003 μM and 60 μM. Drug efficacy (E**_**max**_**) was included as additional measure of response, indicated with light grey to black boxes according to percent efficacy above the waterfall plot. The genotype of each culture according to panel sequencing is color-coded according to the legend given at the bottom of the figure. Area (grey) of achievable steady state *in vivo* plasma concentrations (c**_**ss**_**) are given in μM and indicated with grey dotted lines. (**A-C**) IC**_**50**_ **values for small molecules gefitinib, afatinib and sapitinib, targeting the ERBB receptor(s) ERBB1/EGFR, ERBB2/Her2, ERBB3 and ERBB4. (**D**) Inhibition at the level of MEK1/2 with selumetinib. (**E**) Response to the multikinase inhibitor regorafenib. (**F**) Treatment with the mTORC1/2 inhibitor BI-860585. The *BRAF***^**G466R**^ **and *BRAF***^**G466V**^ **mutations in tumors OT170 and OT327, respectively, are not included in the figure, as their gene products are considered kinase-dead [77]**.

For anti-EGFR treatment testing of 38 *KRAS*-mutant and wild-type organoid cultures, we focused on the small molecules gefitinib, afatinib, and sapitinib (**Fig 2A–2C**, **S3** and **S4 Tables**) rather than on the monoclonal antibody cetuximab because its effects in cell culture do not correlate well with clinical action. Earlier reports described IC_50_ values for cetuximab in the millimolar range, either directly measured or extrapolated [9, 26, 27], while biologically relevant concentrations were reported to be in the nanomolar range [28]. In addition to preventing the binding of ligand to its receptor, cetuximab exerts antibody-derived cellular cytotoxicity, not recapitulated well under cell culture conditions [29, 30].

We considered potentially relevant inhibitory effects of the compounds at a range of concentrations achievable in patient plasma—i.e., at or close to the c_ss_ steady-state concentration (**Fig 2**, grey areas) that reduced viability of >50% of cells under the testing conditions. The IC_50_ values for gefitinib ranged from 0.12 μM to >60 μM. Thirty-one of 38 cultures were resistant to gefitinib, including 13 quadruple-negative cultures (*KRAS*^wt^, *NRAS*^wt^, *BRAF*^wt^, and *PIK3C*A^wt^) and 16 cultures harboring mutations in at least one of these critical genes. Four quadruple-negative, two *PIK3CA*-mutant, and one exceptional *BRAF*^V600E^-mutant cultures responded to gefitinib treatment. The resistance to receptor tyrosine kinase inhibitors corroborated previous preclinical and clinical findings [4], although the range of growth responses indicated substantial heterogeneity of cultures regardless of critical driver mutations. The IC_50_ values for afatinib and sapitinib ranged from 0.025 μM to 11.7 μM and from 0.033 μM to >60 μM, respectively. In total, 34 cultures did not respond to afatinib, and 25 did not respond to sapitinib.

**Fig 3:**
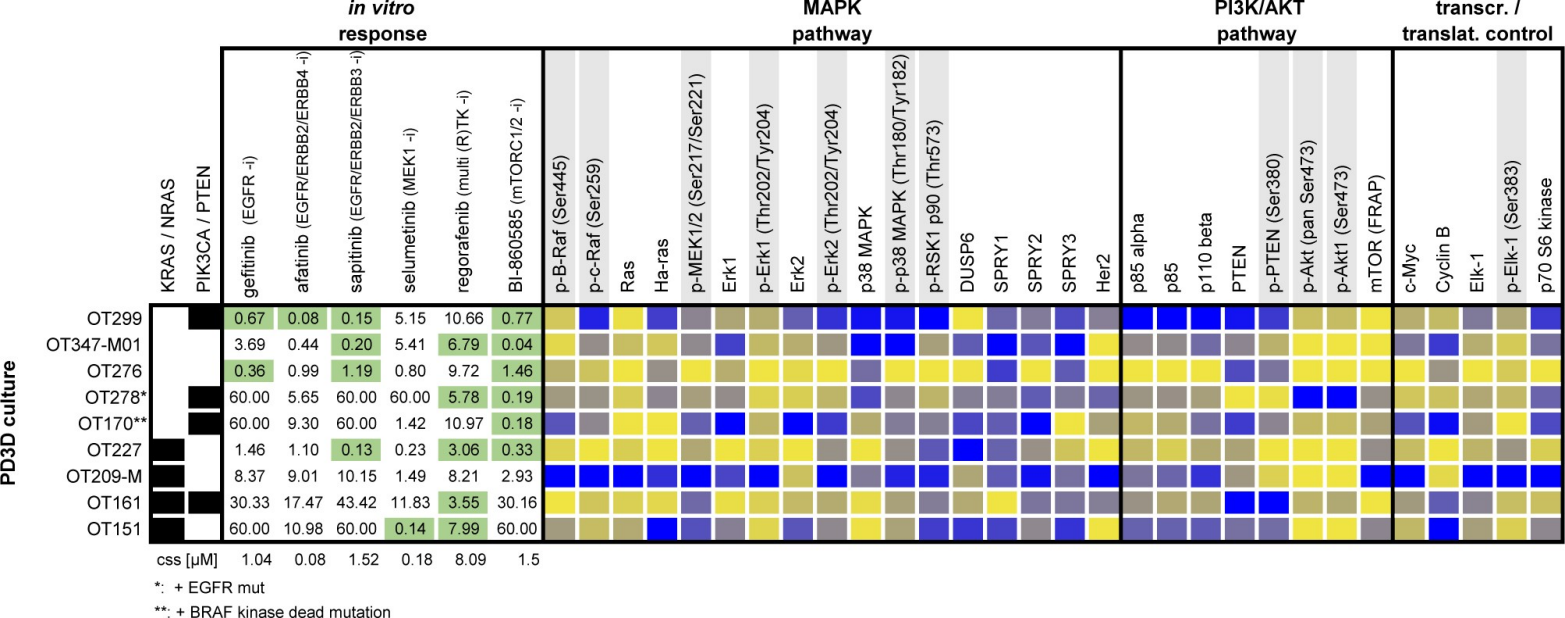
Bead-based western blot analysis of (phospho-)proteins in 9 organoid cultures. DigiWest-based protein expression analysis of 30 (phospho-) proteins in 9 PD3D cultures is shown together with genotype and response data. Samples are sorted according to *RAS* mutation status. Individual IC_50_ values are shown for tested compounds. Green marked IC_50_’s indicate susceptibility to the respective small molecule. Protein expression values are column-wise color-coded from lowest (yellow) to highest (blue) expression for each analyte. Phosphoproteins are marked in grey.

In view of the heterogeneous drug response, we added another layer of molecular information to the models by contrasting protein profiles of nine organoid cultures, matched tumor tissues, and morphologically normal tissues adjacent to the tumors (**S2 Fig**, **S5 Table**). We applied the novel DigiWest bead-based western blot method, designed for in-depth protein profiling of cellular signal transduction [24]. Overall, protein and phosphoprotein abundance varied substantially among cultures and tissues. Next, we correlated the drug response with the activation status of the MAPK pathway (**Fig 3**). Surprisingly, gefitinib-resistant cultures OT227 (*KRAS*^G13D^), OT209-M (*KRAS*^G12V^), OT161 (*KRAS*^G12S^), and OT151 (*NRAS*^Q61K^) exhibited very diverse pathway activation, ranging from throughout high expression of p-ERK1, p-ERK2, and pRSK1, for example, to low expression and phosphorylation, respectively. Expression of DUSP6, an antagonist of MAPK signaling involved in feedback regulation, was not consistently associated with diminished phosphorylation of signaling kinases. We observed a similarly diverse pattern of pathway activation in gefitinib-responsive *RAS*^wt^ organoids OT299 and OT276 and the resistant cultures OT347-M01 and OT278.

In addition to blocking receptor tyrosine kinase activity, we treated organoid cultures with the MEK inhibitor selumetinib, the multi-kinase inhibitor regorafenib, and a novel mTORC1/2 inhibitor BI-860585 [31] (**Fig 2D–2F**, **S3** and **S4 Tables**). We found resistance to selumetinib in 16 out of 17 quadruple-negative organoids and in 13 out of 16 *RAS*-mutated ones. Three *PIK3CA*-mutated, *RAS*^wt^ organoid cultures were equally resistant, while the *BRAF*^V600E^ cultures OT261 and OT212 were sensitive. This finding would suggest that in the absence of damaging *RAS* and *PIK3CA* mutations, signaling downstream of BRAF was efficiently blocked. The two *KRAS*^G12D^ cultures OT302 and OT288 were exceptionally sensitive to MEK inhibition; however, the susceptibility towards the inhibitors was not exclusive for this kind of *KRAS* mutation.

Treatment with the multi-kinase inhibitor regorafenib that targets CRAF and VEGF receptors resulted in responses irrespective of the mutational status of *RAS* and *PIK3CA*, with the distribution of sensitive versus resistant cultures being approximately equal for wild-type and mutant cultures (**Fig 2E**). The range of IC_50_ values in most cultures was relatively narrow (3.05–23.9 μM), except for cultures OT114 and OT152 (>60 μM) and OT234 (0.58 μM). Since regorafenib specifically targets the tumor vasculature, any conclusions regarding the proliferation of organoids are very limited [14].

Nineteen out of 38 organoid cultures responded upon treatment with BI-860585, and 19 were resistant (**Fig 2F**). All *PIK3CA/PTEN*-mutant, *KRAS*^wt^ organoids responded to the mTORC1/2 inhibitor, three *PIK3CA/KRAS* mutant cultures were resistant, and two of them were sensitive. Analysis of protein extracts by the bead-based western blotting method revealed that neither the level of p85 (alpha), p110 beta or PTEN expression nor the phosphorylation status of PTEN and AKT correlated with the response to mTORC1/2 inhibition (**Fig 3**).

Next we analyzed the effects of the RTK inhibitors gefitinib, afatinib and sapitinib by profiling 3 selected organoids OT151, OT276 and OT347-M01 employing DigiWest protein expression profiling after drug exposure for 72h. To avoid substantial cell loss and accumulation of cellular debris during drug exposure, we chose drug concentrations primarily based on the clinically achievable plasma concentrations (c_ss_, **S3 Table**). There were two exceptions, in which the IC_50_ was achieved already below the relevant plasma concentration in patients. Accordingly, we treated OT276 with 0.35 μM gefitinib and OT347-M01 with 0.20 μM sapitinib. The detailed results of DigiWest analysis are shown in **S3 Fig** and **S6 Table**. Hierarchical clustering of the analyzed data showed that patient derived organoids formed three separate branches in the dendrogram. These were split further into branches distinguishing gefitinib from afatinib and sapitinib treatment indicating a dominant but distinctive effect of the tested drugs on each individual organoid culture.

For example, the treated *NRAS* mutant organoid OT151 showed an increase in phosphorylation of central proteins of the MAPK signaling cascade, including ERK, RSK, and S6 ribosomal protein **(S3 Fig, S6 Table)**. While this expression- and activity pattern could easily be reconciled with the resistance to anti-receptor tyrosine kinase inhibition, there was no clear correlation between treatment resistance and drug-modulated signaling patterns in OT276 and OT347-M01 cells. OT276 cells were sensitive to afatinib and sapitinib treatment although p-MEK was increased. Sapitinib sensitivity in OT347-M01 cells was associated with low MAPK activity. We observed a distinct modulation of WNT signaling indicated by GSK3β phosphorylation and a high increase in active beta-catenin in OT347-M01 cells. MTOR activity appeared to be high in OT276 but low in OT347-M01. Overall, most alterations observed 72h after drug exposure are very likely due to the rewiring of the entire signaling system involving adaptive feed-back and cross-talk mechanisms.

### Genetic, transcriptomic, and inhibitor response heterogeneity in multi-sampled cell culture models derived from a single primary tumor

**Fig 4:**
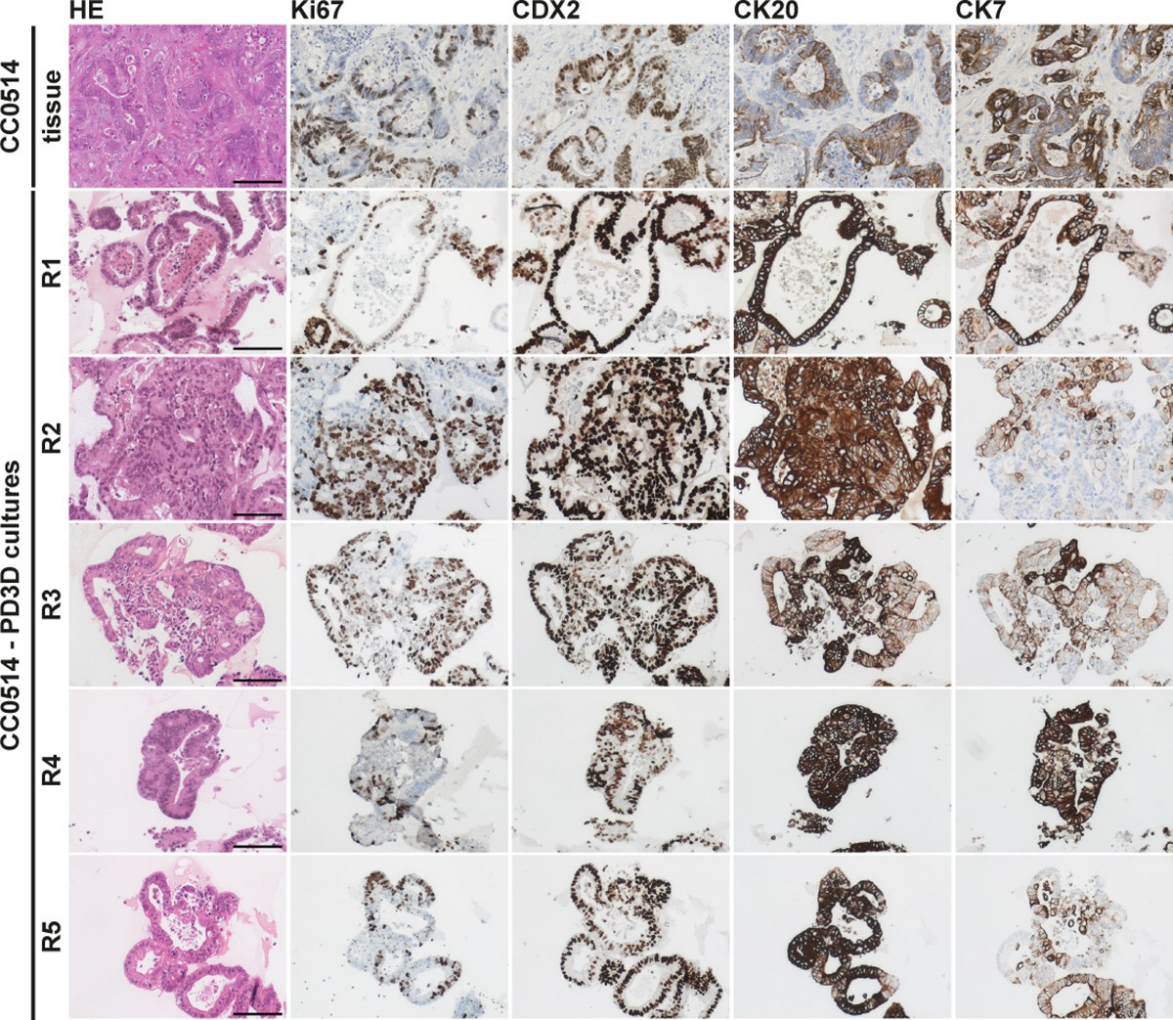
Immunohistochemical stainings of “sibling organoids” and original colorectal carcinoma. Immunohistochemical staining with hematoxylin and eosin (HE) and expression of Ki67, CDX2, CK20 and CK7 show overlapping patterns in tumor tissue and derived PD3D cultures R1-R5 of patient CC0514. Scale bars: 200 μm.

To investigate the impact of ITH on treatment effects in organoid cultures, we analyzed mutational patterns and drug responses of five organoid “sibling” cultures established in parallel from separate regions of an individual primary colon carcinoma (CC0514-R1, -R2, -R3, -R4, and -R5). Tissue architecture, Ki67 expression, and expression patterns of markers routinely used to characterize tumors of the colonic epithelium recapitulate those of the donor tumor (**Fig 4**). Using the 50-gene cancer panel, we detected common *KRAS*^G12D^, *PIK3CA*^H1047R^, and *TP53*^C242F^ mutations in the tumor tissue-of-origin and the separate sibling cultures, as well as an additional homozygous *SMAD4*^R361H^ mutation in cultures CC0514-R1 and CC0514-R2 (**Fig 1B**, **S2 Table**). This mutation affects the MH2 domain of the protein and abolishes R-SMAD/SMAD4 heterodimerization [32]. All sibling cultures represented the molecular subtype CMS2 (**S7 Table**), according to the classification by Guinney and colleagues [3].

**Fig 5:**
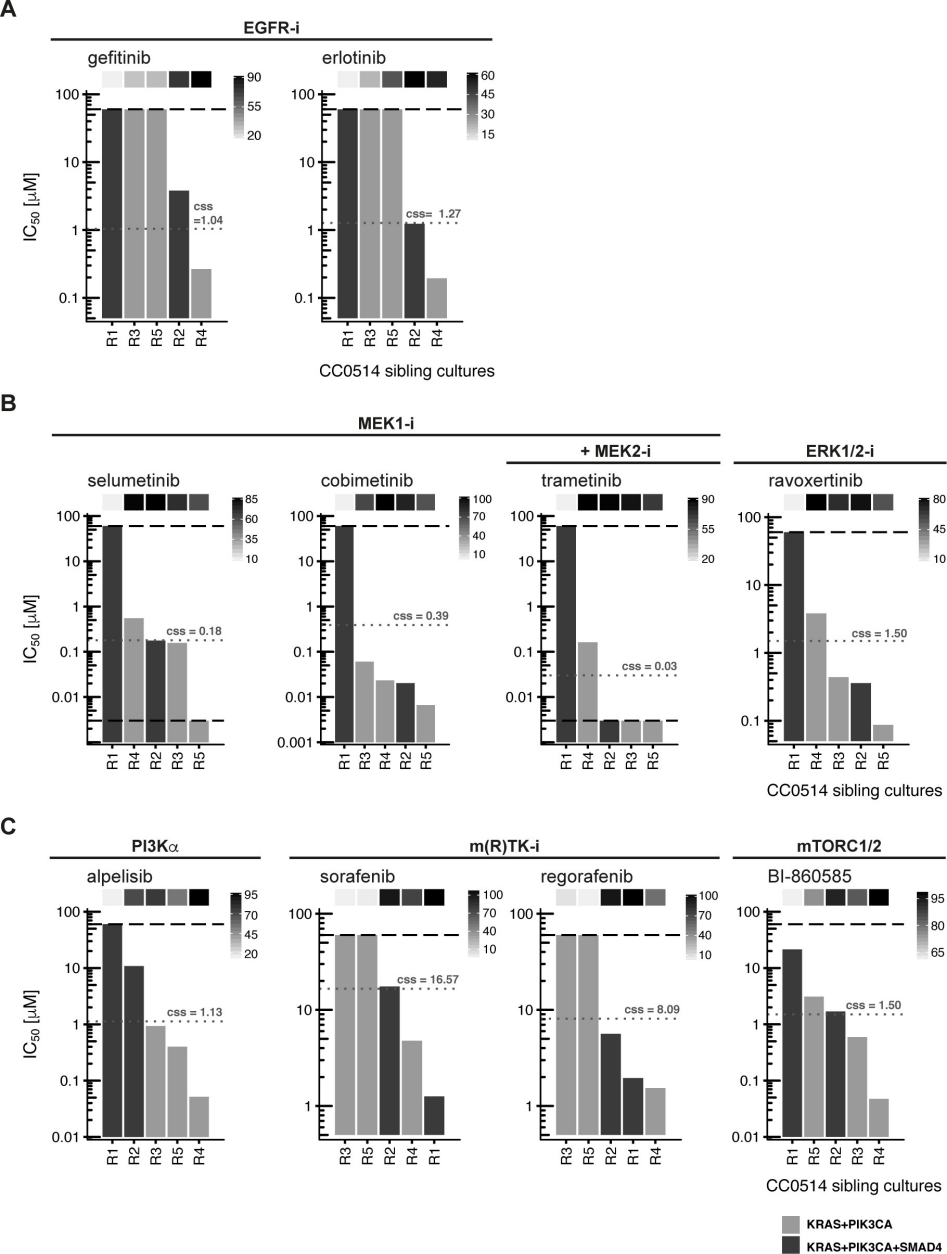
Organoid sibling cultures display heterogeneous drug responses. **IC**_**50**_ **values for sibling cultures derived from patient tumor CC0514 treated with a panel of small molecules targeting EGFR, MEK/ERK, PI3Kα, mTORC1/2 and multiple kinases are shown. The lower and upper assay cutoffs were 0.003 μM and 60μM. Drug efficacy (E**_**max**_**) was included as additional parameter of response, indicated with light grey to black boxes above the waterfall plot according to percent efficacy. Critical mutations in each culture are depicted in light and dark grey bars according to the legend at the bottom of the figure. Achievable steady state *in vivo* plasma concentrations (c**_**ss**_**) are given in μM and indicated with grey dotted lines. (**A**) IC**_**50**_ **values found following inhibition at the EGF receptor. (**B**) IC**_**50**_ **values found following inhibition at downstream pathway components MEK and ERK. (**C**) Inhibition with alpelisib (targeting PI3Kα), BI–860585 (mTORC1/2) and the multi-kinase inhibitors sorafenib and regorafenib**.

First, we assayed drug responses in the sibling cultures using inhibitors targeting EGFR, MEK, ERK, p110α, and mTORC1/2 as well as sorafenib and regorafenib (**Fig 5A–5C**, **S4 Table**). The drug responses of sibling cultures were not uniform. Organoid culture R1 was unique by being unaffected by inhibitors targeting EGFR, MEK, ERK, PIK3CA, and mTORC1/2. In contrast, culture R4 was sensitive toward EGFR, PI3Kα, and mTORC1/2 inhibition. The remaining cultures, R2, R3, and R5 shared resistance with R1 to EGFR inhibition, but responded heterogeneously to the other compounds.

**Fig 6:**
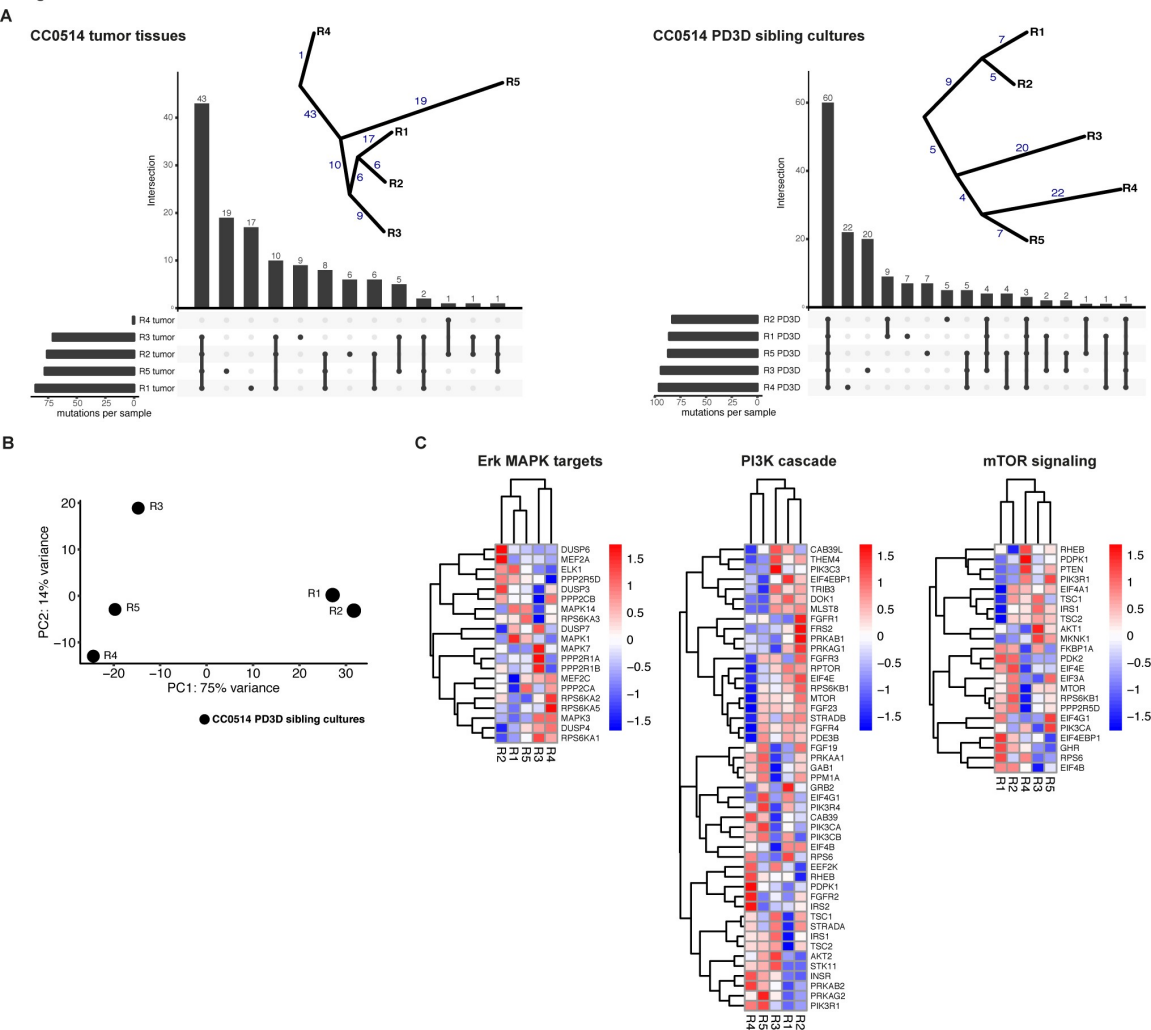
Tumor tissues and sibling cultures of patient CC0514 display genetic heterogeneity and heterogeneous mRNA expression profiles. **Heterogeneity of tumor tissues and PD3D sibling cultures was evaluated on DNA and mRNA levels. (**A**) On the genomic level, somatic mutations were called from DNA of the five original tumor pieces (R1-R5) of the primary tumor of patient CC0514 and the respective PD3D sibling cultures, compared to CC0514 patient´s blood. Cellular content of tumor tissue R4 was very low. UpSet plots show rare somatic mutations (MAF < 0.001) in exonic regions used to construct evolutionary trees of the somatic mutations, displayed next to the plots. The numbers of shared or private mutations are given. (**B**) Principal Component Analysis of the mRNA expression of the sibling cultures. First component on x-axis contains 75% of the variance and classifies the samples into two major groups R1/R2 vs. R3/R4/R5). (**C**) Heatmaps of mRNA expression of components of ERK/MAPK, PI3K and mTOR signaling pathways. Each row has been transformed using Z-score. The color code represents the scaled mRNA expression across samples. Genes and samples were hierarchically clustered using Euclidean distance**.

As the underlying mutations did not sufficiently explain the observed differences in drug response, we expanded molecular analysis by exome and RNA sequencing. Exome sequencing confirmed the mutations found via panel sequencing. An additional shared heterozygous mutation was found for beta-catenin (*CTNNB1*^R582W^). The original tumor tissues and sibling cultures exhibited a remarkable genetic heterogeneity (**Fig 6**). Unbiased analysis of their somatic mutation landscapes highlighted specific mutations in every single region or derived culture (**Fig 6A**, **S8 Table**). Phylogenetic trees of the tumor mutations suggested a grouping of tumor regions and organoids CC0514-R1/R2 versus R3, R4, and R5. Moreover, unbiased principal component analysis (PCA) based on the complete transcriptomes of the cultures showed a high variance between cultures R1/R2 and R3, R4, and R5 (**Fig 6B**). Overall, we identified 646 differentially expressed genes when contrasting *SMAD4*^R361H^ and *SMAD4*^wt^ cultures (R1 and R2 vs. R3, R4, and R5; adj. *p* < 0.05, logFC >1, **S9 Table**). We visualized the mRNA transcriptome of sibling cultures in heatmaps, focusing on ERK/MAPK-, PI3K-, and mTOR-signaling pathways (**Fig 6C**). Although retrieving the same sample dichotomy between the R1/R2 and R3–5 groups, we observed substantial heterogeneity in mRNA expression of target genes encoding components of the respective pathways.

### *In vivo* modeling of intratumor heterogeneous cellular expansion and treatment response

**Fig 7:**
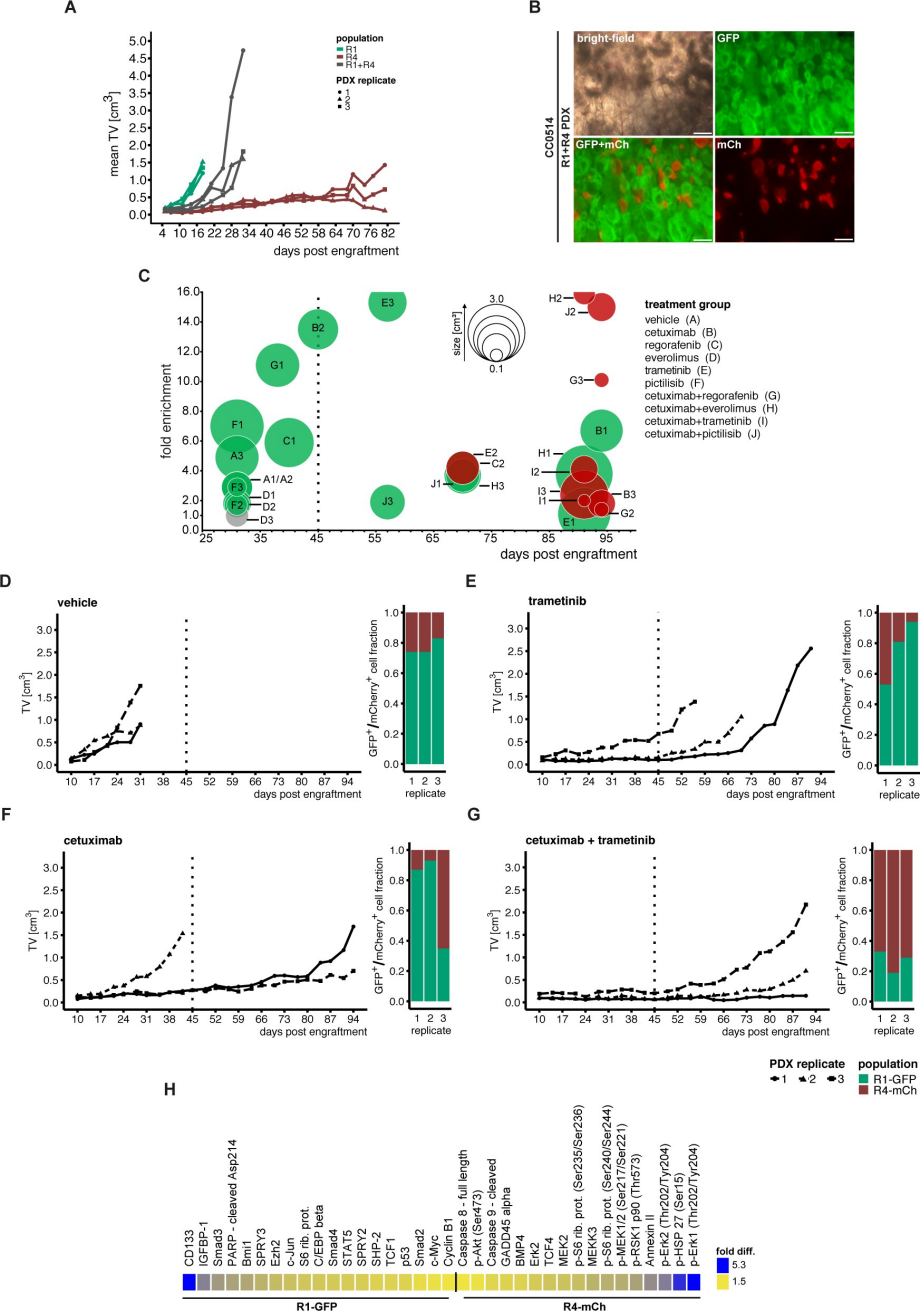
Subpopulation-specific response to *in vivo* drug treatment. **(**A**) Cells of sibling cultures CC0514-R1 and CC0514-R4 were transduced with PKF-GFP and PGK-mCherry (mCh) markers, respectively. 1.0×10**^**6**^ **cells were injected into nude mice either as single populations (green/red) or as a 1:1 mixture of both populations (grey) in triplicate. (**B**) Microscopic images of a mixed R1-PGK-GFP and R4-PGK-mCherry tumor, scale bars: 200μm. (**C**) Mixed populations of R1 and R4 cultures were subjected to treatment *in vivo* in triplicate. Treatments started 10 days post injection included 5 single compounds and combinations with cetuximab. Treatments were carried out until 45 days (dashed line), if possible. PDX tumors showing minor growth post treatment were maintained *in vivo* to monitor subsequent growth. The bubble plot shows tumor sizes, as represented by bubble diameter, and fold enrichment of cell subpopulations for all replicates in treatment groups A-J (displayed in the figure). Green (= GFP**^**+**^**) or red (mCherry**^**+**^ **= mCh**^**+**^**) fills indicate which subpopulation was more abundant in the PDX tumor, as measured by FACS analysis of re-suspended tumor cells. Grey circles indicate a 50:50 distribution of labelled tumors. Note that for PDX tumors C2 and E2 both fold enrichment and tumor size were at a similar range** (S11 and S12 Tables)**. (**D-G**) Tumor growth kinetics during the course of the *in vivo* mixing experiment are shown together with the fractions of GFP**^**+**^ **and mCh**^**+**^ **populations at the end of the experiment (FACS analysis). Treatments were done with vehicle, trametinib, cetuximab and cetuximab+trametinib combination. (**H**) Protein expression of CC0514-R1-GFP and CC0514-R4-mCh organoids analyzed by DigiWest protein assay. Difference in fold expression ranging from 1.5 (yellow) to 5.3 (blue)**.

To model the therapy response in a heterogeneous tumor cell population *in vivo*, we first labeled organoid cultures CC0514-R1 and CC0514-R4 by transduction with phosphoglycerate kinase (PGK) promoter-driven expression vectors encoding the fluorescence markers GFP and mCherry (mCh), respectively. We then prepared single-cell suspensions of R1-GFP and R4-mCh cells embedded in Matrigel, separately or as a 1:1 mixture of R1-GFP and R4-mCh cells, injected them subcutaneously into nude mice, and calculated the tumor volumes over time (**S10 Table**). The R1-GFP cells rapidly formed tumors with a mean volume of 1.36 (±0.16) cm^3^ after 18 days, while the R4-mCh tumor xenografts achieved less than one-tenth of these volumes (mean 0.11 cm^3^ ±0.03) after the same period. Even at >80 days after inoculation, R4-mCh tumors were smaller (mean volume 0.76 cm^3^ ±0.66) than R1-GFP tumors that had formed much earlier. The mixed suspensions of R1-GFP and R4-mCh cells produced tumors after a short lag phase. Tumor volumes comparable to the ones produced only by R1-GFP cells were reached approximately 10 days later (**Fig 7A**, **S10 Table**). Notably, the architecture of mixed-culture xenografts indicated strict clonal outgrowth *in vivo*, as the R1-GFP and R4-mCh cells always formed separate organoid structures (**Fig 7B**).

For targeting the EGFR/RAS/MEK-signaling system *in vivo*, we chose the clinically approved EGFR antibody cetuximab and the MEK inhibitor trametinib (**S11 Table**). The duration of treatment with individual and combined inhibitors was planned for 36 days, following an initial period of 10 days post-injection to achieve engraftment. However, in cases of rapid tumor establishment and progression, the experiment had to be terminated earlier. After measuring tumor volumes, we sacrificed the mice, dissociated the tumor cells, and determined the ratio of R1-GFP and R4-mCh cells by FACS (**S12 Table**).

In xenografts derived from mixed cultures, R1-GFP cells formed the dominant population, indicated by its greater potential for expansion (**Fig 7C**, **S10 Table**). Compared to inoculation of single-cell suspensions, co-injection of R1-GFP and R4-mCh cells, left untreated or treated with vehicle, retarded the onset of tumor growth slightly, and progression to 1.0 cm^3^ tumors was apparent after an additional week (**Fig 7D**). Treatment of the *KRAS*-mutant mixed cultures with cetuximab or trametinib alone reduced tumor take rate (determined on day 38 post-injection) and substantially prolonged the period required for achieving an equal 1.0 cm^3^ tumor mass in two out of three tumors (**Fig 7E** and **7F**). At the end of this experiment, the R1-GFP population outnumbered the R4-mCh population in controls and inhibitor-treated tumors. Reduction of tumor takes and volumes was more pronounced following cetuximab/trametinib co-treatment. Moreover, the R4-mCh population outnumbered the R1-GFP population, indicating that the latter population was vulnerable to co-treatment, although a minor fraction of R1-GFP cells survived combination treatment (**Fig 7G**, **S12 Table**). The initial response to cetuximab treatment of R1-GFP tumors may be due to a surprisingly low activation level of MAPK signaling; however, antibody-dependent toxicity cannot be excluded (**Fig 7H, S5 Table**). In contrast, the R4-mCh population exhibited enhanced levels of p-ERK1/2 in spite of its low growth potential *in vivo*. The growth kinetics of R4-mCh organoids *in vivo* suggest that the duration of inhibitor exposure until day 45 post-injection into mice was too short for this slowly expanding population, although *in vitro* testing showed susceptibility to anti-EGFR treatment (**Fig 5**). The presence of GFP-positive cells beyond day 45 suggested that after initial debulking, this population escaped from treatment by anti-receptor tyrosine kinase antagonists, in-line with the results from *in vitro* testing. The slowly expanding R4-mCh cells may even have provided a niche for surviving R1-GFP cells.

The R1/R4 xenografts responded partially to administration of everolimus, regorafenib, and pictilisib, as well as to combinations of these three drugs with cetuximab (**S11 Table**). In five out of six experiments, the proportion of residual R1-GFP cells relative to R4-mCh cells after treatment indicated partial response of the rapidly proliferating R1-GFP population and the relative resistance of the R4-mCh population able to increase in number at the expense of R1-GFP cells.

## Discussion

Here we show heterogeneous pathway activity and inhibitor responses in CRC organoid cultures profiled by cancer gene panel sequencing and DigiWest-based signaling-pathway analysis. Eight *KRAS* ^G12V/S/D^ cultures were resistant to EGFR inhibition by gefitinib, as were four *KRAS*^G13D^ and three *NRAS*^Q61K^ cultures, one *KRAS*^A146T^ and one *BRAF*^V600E^ culture. Four quadruple-negative and two *RAS* wild-type/*PIK3CA*-mutant cultures responded to gefitinib. Basically, these results are in line with diagnostic and clinical experience regarding the administration of therapeutic antibodies targeting EGFR [4] and a recent report on colorectal cancer organoids [15]. In our cohort, 12 quadruple-negative cultures were resistant to gefitinib treatment. The magnitude of drug resistance observed in quadruple-negative cultures is reminiscent of clinical experience in tumor treatment. For example, the organoid culture OT330 and its donor tumor harbored an *ERBB2* amplification [11]. High ERBB2 expression detected *in vitro* is known to cause resistance to EGFR-targeting compounds [33, 34]. Moreover, OT330 shared increased levels of MET mRNA with the *KRAS*^wt^ cultures OT216 and OT281. High MET expression in the absence of activating *KRAS* mutations may mediate primary resistance and an escape mechanism from anti-EGFR receptor treatment [35].

Since extracellular signal-regulated kinases (ERKs) control the biological outcomes of EGFR/RAS signaling [36], we analyzed their activation status in organoid cultures. Interestingly, the presence of *KRAS/NRAS* mutations did not robustly coincide with MAPK pathway activation determined by the enhanced steady-state phosphorylation of effector kinases BRAF, CRAF, MEK1/2, ERK1/2, or RSK1 p90. Consequently, the wild-type status of KRAS or NRAS proteins did not predict gefitinib response. Similarly, monitoring the activation status of the MAPK pathway downstream of RAS did not substitute as a diagnostic approach for predicting treatment response. For example, *KRAS*^G12S^ organoid OT161 exhibiting low pathway activation, reminiscent of a “functionally wild-type” status, was gefitinib-resistant. Conversely, *KRAS/NRAS/BRAF*^wt^ culture OT299 exhibited a “functionally mutated,” highly active pathway status in spite of its susceptibility to gefitinib and other EGFR inhibitors. The polyclonal nature of organoids analyzed in early passage most likely reflects the intrinsic molecular heterogeneity of the donor tumor and may even preclude the detection of homogeneously strong MAPK activation. Eventually, subcloning of organoid cultures may allow enrichment of cells exhibiting uniform pathway activation in the presence of the corresponding upstream mutations. However, the value of cloned organoids for serving as avatars for the donor tumor will be diminished, since ITH is no longer maintained. The uncoupling of mutational *KRAS* status and pathway activation is not unprecedented, as shown in pancreatic adenocarcinoma and colorectal cancer [37, 38]. *BRAF*^V600E^ mutant colorectal cancers have been shown to express differential activation of the KRAS/AKT pathway and cell cycle proteins, indicating a diverse biology for a subtype of colorectal cancers even driven by the same driver mutation [39]. Here we show that MAPK signaling activity is diverse and not directly affected by the presence of a *KRAS* mutation. Hence, the true biology of these tumors may only be understood after conducting further comprehensive mechanistic studies. MAPK pathway activation is known to be modulated by dual-specificity phosphatase 6 (DUSP6) and Sprouty (SPRY) isoforms, which constitute transcriptional feedback loops and exert post-translational functions that constrain signaling-kinase activities [40–42]. However, the protein levels of DUSP6 and SPRY1, 2, and 3 were not correlated with MAPK activation in our organoid cultures.

Drug susceptibility testing was done in polyclonal organoids in low passage early after establishment of cultures. It is very likely that tumor subpopulations present in newly established models exhibit different degrees of fitness and pathway activity, a diverse potential to expand, and non-homogenous inhibitor responses. In general, the big-bang hypothesis [20] predicts that during tumor evolution, many subpopulations exhibiting diverse mutation patterns, phenotypes, and treatment responses can emerge. Variable clonal population dynamics can substantially modulate treatment response and tolerance [43]. We investigated this scenario through multi-region sampling from a single colorectal tumor and established sibling organoids. Molecular analysis by sequencing crucial cancer genes, exomes, RNA, and targeted proteomics identified uniform *KRAS*, *TP53*, and *PIK3CA* driver gene mutations in all parallel cultures, but also provided evidence for substantial ITH. Not surprisingly, the sibling cultures showed profound differences in treatment response. Organoid culture R4 responded to 7 out of 10 inhibitors *in vitro*, while R1, harboring an additional *SMAD4* loss-of-function mutation, was resistant to all small molecules tested, except for regorafenib and sorafenib. The response pattern was unequal in R2 organoids despite sharing the *SMAD4* mutation and overall RNA expression pattern. The proliferation potential of R1 and R4 organoids *in vivo* differed substantially in spite of common *KRAS*^G12D^, *PIK3CA*^H1047R^, and *TP53*^C242F^ mutations. During the cetuximab/trametinib treatment cycle in xenografts, R4 cells survived and later formed the majority of the tumor post-therapy. The role of high ERK activation in R4 cells is not clear. High ERK activation does not necessarily drive proliferation, but it can be growth-inhibitory [44, 45].

The homozygous *SMAD4*^R361H^ mutation was not detectable in the bulk tumor CC0514. We assume that acquisition of the mutation was not due to a *de novo* event in culture, but rather was present in a minor subpopulation of the original tumor. The loss of SMAD4 function, known to trigger tumor progression and metastasis [46–48], may explain the high proliferative potential of R1 organoids. In contrast, R4 organoids express presumably growth-inhibitory levels of phosphorylated ERK and resemble a cancer stem cell or progenitor phenotype, as described in Blaj *et al*. [38]. Interestingly, SMAD4 proteins can be degraded via ERK1/2 signaling [49], suggesting that SMAD4 does not function normally, even in poorly proliferative R4 organoids.

In summary, our findings imply that the administration of therapeutics to bulk organoid cultures may fall short of correctly displaying the functional diversity of existing subpopulations, due to heterogeneities of their genomes and transcriptomes. The great challenge of interpreting and reconciling multi-omics and response data in apparently closely “related" tumor cells was recently demonstrated by Roerink and colleagues [50]. The authors investigated organoid cultures established from single cell clones of different tumor areas of colorectal cancer patients. Via genomic sequencing, methylome analysis and RNA sequencing, they reconstructed evolutionary trees with high concordance between (epi)genomics and gene expression data. Yet, drug response in different organoids from the same tumor displayed substantial differences in IC_50_ values of up to 1,000-fold for chemotherapeutic agents and targeted inhibitors. The molecular events that lead to differential drug sensitivity of closely related organoids from the same tumors are currently unknown.

In future diagnostics settings, multisampling of malignant tissues, drug sensitivity testing in culture and the depth of subsequent molecular analysis will have to be carefully balanced under consideration of sample availability, time and even funding constraints. The molecular analysis of organoid cultures in this study was limited by the focus on frequent oncogenes and tumor suppressor genes as well as by the availability of validated antibodies used in DigiWest assays. Notwithstanding such limitations, analyzing the dynamics of sibling cultures may help to overcome the limitations of predicting precision targeting of mutations retrieved by genomic analysis alone [51] and to better inform therapeutic decision-making.

## Materials & methods

### Ethics statement

EPO strictly follows the EU guideline European Convention for the Protection of Vertebrate Animals Used for Experimental and Other Scientific Purposes (EST 123) and the German Animal Welfare Act (revised version Art. 3 G v. 28.7.2014 I 1308). Furthermore, we handle our animals according to the Regulation on the Protection of Animals Used for Experimental or for Other Scientific Purposes (Tierschutz-Versuchstierverordnung- TierSchVersV: revised version Art. 6 V v. 12.12.2013 I 4145). Compliance with the above rules and regulations is monitored by the Landesamt für Gesundheit und Soziales (LAGeSo), which is the responsible regulatory authority monitoring animal husbandry.

### Generation and propagation of organoid cell cultures

Organoid cultures were generated and propagated as previously described [11, 52]. Overall, 86 patient-derived three dimensional (PD3D) cell cultures were generated, including five samples that were isolated from mouse xenografts (PDX) and one sample that underwent transient engraftment and was then reintroduced into an *in vitro* organoid culture (OT151-PDX-PD3D). Additionally, for patient CC0514 we generated five organoid cultures from five separate regions of the primary tumor. The full cohort is described in **S1 Table**.

### Immunocharacterization

Two μm de-paraffinized FFPE tissue sections of donor tumors or organoid cultures grown for five days were stained using the primary antibodies anti-CK7 (clone OV-TL12/30, Dako, Germany), anti-CK20 (clone KS20.8, Dako), anti-CDX2 (clone CDX2-88, BioGenex, U.S.A.), and anti-KI67 (clone MIB-1, Dako) for 32 min at 37°C, using the ultraView DAB detection kit (Ventana, U.S.A.) on the BenchMark XT instrument (Ventana), as recommended by the manufacturer. Counterstaining was performed with Hematoxylin II Counterstain and Bluing Reagent (Ventana) for 4 min. Microscopy was performed with a Zeiss Axiovert 400 microscope (Zeiss, Germany).

For immunofluorescence imaging, organoid cultures were fixed in 4% paraformaldehyde for 30 min at room temperature and permeabilized with 0.1% Triton X-100 for 30 min. Samples were blocked in phosphate-buffered saline (PBS) with 10% bovine serum albumin (BSA) and incubated with primary antibodies overnight at 4°C. Antibodies used were Anti-Ezrin (clone EP886Y, Abcam, diluted 1:200) and EPCAM (VU1D9, Cell Signaling Technology, diluted 1:500). Samples were stained overnight with a conjugated secondary antibody at 4°C. F-actin was stained with Alexa Fluor 647 Phalloidin (#A22287, Thermo Fisher, diluted 1:20) for 30 min at room temperature. Nuclei were counterstained with DAPI (Sigma Aldrich). Cells were then transferred to microscope slides for examination using a Zeiss LSM 700 laser scanning microscope.

### Nucleic acid preparation

Genomic DNA from organoid cultures and fresh tumor tissues was prepared using the AllPrep DNA/RNA Kit (QIAGEN, Germany) according to the manufacturer’s protocols, and it was quantified using a Qubit 2.0 Fluorometer and the appropriate assay kits (Life Technologies, Germany). Genomic DNA from FFPE material from at least three consecutive 10 μm sections was isolated using the QIAGEN QIAamp DNA FFPE Tissue Kit to obtain sufficient amounts for targeted panel sequencing. The yields of FFPE-derived DNA were quantified with the TaqMan RNase P detection assay (Life Technologies).

### Targeted deep sequencing and microsatellite status

Targeted sequencing was performed on an IonTorrent PGM bench-top sequencer (Life Technologies). Ten nanograms of genomic DNA were used to prepare Ion AmpliSeq Cancer Hotspot Panel v2 (Life Technologies) amplicon libraries in conjunction with the Ion AmpliSeq Library Kit 2.0 (Life Technologies). For multiplexing purposes, unique Ion Xpress barcode adapters were assigned to every sample. For amplification, 17 or 20 PCR cycles were applied for cell-culture-based and FFPE-tissue-based samples, respectively. Amplicon libraries were quantified with the Ion Library Quantitation Kit (Life Technologies), diluted to 100 pM, and pooled in an equimolar concentration. Clonal amplification of single PCR templates was carried out on an Ion OneTouch 1 cycler in conjunction with the Ion OneTouch 200 Template Kit v2 DL. Sequencing of four to five pooled libraries was performed using Ion 316v2 sequencing chips. Base calling, read mapping, and coverage analysis were performed with the default settings of Torrent Suite software version 4.0.2. Targeted panel sequencing yielded over 44.0×10^6^ mapped reads, and the oversampling rates averaged to 3.6×10^3^-fold. The mean uniformity of coverage (i.e., the distribution of reads across all 207 pooled PCR products per sample) was above 98%. Primary data analysis and variant calling were performed with the built-in Variant Caller tool (version v4.0-r76860), set to the “somatic, high stringency” option and down-sampling to 2,000 reads. Variant calls were visually inspected using the Integrated Genomics Viewer (IGV) [53] and annotated in accordance with HGVS recommendations [54]. To assess the effect of the retrieved mutations, the prediction tools SIFT [55], PolyPhen2 [56], and MutationTaster2 [57] were used. Visualization of mutations was carried out using the R Script ComplexHeatmap [58].

Additional Sanger sequencing was performed for sibling cultures CC0514-R1, CC0514-R2, and CC0514-R5 as early as six weeks post-culture initiation (**S5 Fig**), confirming the panel-sequencing results found for regions R1 and R2 (both *SMAD4*^R361H^) and R5 (*SMAD4*^wt^).

We also compared the mutation information gathered via panel sequencing with whole genome and whole exome data generated for the recently published study by Schuette *et al*. [11].For an overlap of 40 organoid cultures for both methods and overlapping genes, 78% (103/132) of the mutations were covered by the targeted sequencing approach, while 22% (29/133) of the mutations found with exome sequencing were not covered by the panel’s PCR amplicons (**S13 Table**). These included mutations in *APC* (86%, 25/29), *PIK3CA* (2/29), *TP53*, and *PTEN* (1/29 each). Microsatellite status was analyzed as previously described [11, 59].

### Semi-automated high-throughput drug response assays

To perform systematic and parallel testing of drug responses *in vitro*, the organoid culture system was adapted to a 384-well microtiter plate format, as shown earlier [60]. In short, the assay system is based on a luminescence readout of cell viability, measured via ATP consumption. The population doubling time was determined by time-course based measurements using CellTiter-Glo luminescent cell viability assessment. Treatment duration covered two doubling times of the individual cultures, and small molecules were tested at concentrations ranging from 3.0 nM up to 60.0 μM (assay cutoff), as previously shown [11]. Treatment duration of organoid sibling cultures derived from patient CC0514 was uniformly 72 h (**S4 Table**). The half-maximal inhibitory concentration of a compound determined *in vitro* (IC_50_) is a measure of the potency of the compound in blocking cell growth and survival. To assess the potential clinical relevance of the drug response assays, we compared the relative IC_50_ values determined *in vitro* for 43 organoid cultures (**S4 Table**) with steady-state plasma concentrations (c_ss_) of the therapeutic compounds achievable in patients (**S3 Table**). We used reference data obtained from publications or the investigator’s brochures (IB) available at clinical-trial centers. For compounds that have not yet entered late-phase clinical studies, we deduced c_ss_ values from *in vivo* mouse studies (BI-860585) or early (phase I) clinical studies (alpelisib, ravoxertinib). In addition, we determined the maximum inhibition (efficacy, E_max_ (%)) per drug per organoid culture model.

### Whole transcriptome sequencing (RNAseq)

RNA from all organoid sibling cultures of patient CC0514 was extracted and quantified as described above. Whole transcriptome sequencing was performed at the Genomics and Proteomics Core Facility (GPCF) of the German Cancer Research Center (DKFZ). An average of 2 μg total RNA per sample was used to generate barcode-labeled libraries using the Illumina TruSeq RNA sample preparation kit (Illumina, San Diego, CA, U.S.A.). Sequencing of 125 bp paired-end reads was performed on an Illumina HiSeq2000 sequencer with equal distribution of pooled libraries over two sequencing lanes. Mapping to the GRCh37 genome was performed with a STAR aligner [61], allowing maximally two mismatches/alignment gaps and 0.3% total mismatches per alignment. On average, RNAseq yielded 5.1×10^7^ mapped reads (95% unique) covering 5.9 GB coding bases. Reads covering exonic regions per gene ID were counted using HTseq [62]. Downstream analysis was performed using AnnotationDBi, DESeq2 [63], limma [64–66], ggplot2 [67], and GSEA [68].

### Whole exome sequencing (WES)

In total, 11 WES samples were processed, including a blood sample, five separate primary tumor regions from CC0514, and their respective organoid sibling culture derivatives. Whole exome sequencing was performed by the Genomics and Proteomics Core Facility (GPCF) at the German Cancer Research Center (DKFZ). The Agilent SureSelectXT Human All Exon V5 kit was used to generate 125 bp paired-end libraries subsequently sequenced on an Illumina HiSeq2000. The WES pipeline was organized as follows: First, poor-quality reads were filtered out using Trimmomatic [69], and the remaining reads were mapped to the GRCh37 (hg19) human reference genome using the BWA aligner [70]. Afterwards, the Genome Analysis Toolkit [71] was employed for duplicate removal, indel realignment, and base-quality recalibration. On average, 9.5×10^7^ reads were uniquely mapped to the reference genome. We called germline, somatic, and loss of heterozygosity (LOH) mutations using the blood sample as reference with Varscan2 [72]. The minimum coverage for calling variant reads was set to 8 and minimum variant frequency to 0.09. We set tumor purity to 0.5 and minimum tumor frequency to 0.09. Rare SNP and indel mutations were selected as exonic, non-synonymous mutations with an ExAC [73] MAF score below 0.001. Evolutionary trees were built on all somatic mutations in exonic regions for cultures R1–R5. A neighbor-joining algorithm [74], implemented in the “APE” R package [75], was used on the Euclidean distance matrix generated from the binary mutation matrix. We used an artificial null-mutated control as root of the tree. In case of very low tumor cellularity, exome sequencing identified fewer somatic mutations (e.g., in tumor CC0514-R4). Following high-coverage panel sequencing, low-frequency mutations were detected after manually inspecting the sequencing reads (see mutation frequencies for CC0514-R4 tissue in **S2 Table**).

### DigiWest multiplex protein profiling

Tissue samples and organoid cultures were subjected to multiplex protein profiling analysis of up to 150 (phospho-)proteins. In addition, three organoid cultures were subjected to drug treatment prior to protein extraction. For this, 2.4 x 10^5^ cells (= 4 x 10^4^ cells/well in 6 wells total) per condition were plated, followed by a growth period of 72h and subsequent treatment with gefitinib, afatinib, sapitinib and vehicle control (0.03% DMSO) for the duration of 72h. Drug concentrations were chosen based on clinically achievable plasma concentrations (c_ss_, **S3 Table**). In order to monitor the pathway activity status and to gather a sufficient amount of cells for subsequent DigiWest analysis after drug treatment, IC_50_ values were selected for the gefitinib treatment of OT276 (IC_50_ = 0.35 μM versus c_ss_ = 1.04 μM) and sapitinib treatment of OT347-M01 (IC_50_ = 0.20 μM versus c_ss_ = 1.52 μM). After treatment, collected organoids were washed with ice cold PBS, sedimented, treated with Cell recovery solution (Corning) on ice for 30 min, washed twice with ice cold PBS, pelleted and snap frozen at -80°C until cell lysis.

The NuPAGE SDS-PAGE gel system (Life Technologies) was used to separate cellular lysates and blotting. Proteins (20 μg per sample) were fractionated by electrophoresis through 4–12% Bis-Tris gels according to the manufacturer’s instructions. Blotting onto PVDF membranes (Millipore) was performed under standard conditions. For high-content western analysis, the DigiWest procedure was performed as previously described [24]. Briefly, proteins immobilized on the blotting membrane were biotinylated (NHS-PEG12-Biotin, Thermo Scientific), and individual sample lanes were cut into a comb-like structure (strip height = 0.5 mm each, strip width = 6 mm) using an electronic cutting tool (Silhouette SD). The resulting 96 strips corresponded to 96 molecular weight fractions immobilized on individual membrane strips; they covered a range from 15 kDa to 250 kDa.

For protein elution, individual strips were placed in separate wells of a 96-well plate and incubated for 2 hours in 10 μl elution buffer (8 M urea, 1% Triton-X100 in 100 mM Tris-HCl, pH 9.5) for solubilization. After adding 90 μl of dilution buffer (5% BSA in PBS, 0.02% sodium azide, 0.05% Tween-20), 96 different Neutravidin-coated Luminex bead sets (60,000 beads/well) were added to the individual wells, and biotinylated proteins were captured on the bead surface. After overnight incubation, the Luminex beads were pooled, washed, and stored in a storage buffer (1% BSA, 0.05% Tween-20, 0.05% sodium azide in PBS) at 4°C. For antibody incubation, an aliquot of the bead pool (approximately 0.3% of the available bead pool) was transferred into an assay plate, and 30 μl of diluted western blot antibody in assay buffer (Blocking Reagent [Roche Applied Science], 0.05% Tween 20, 0.02% sodium azide, and 0.2% milk powder) was added per well. The list of antibodies is shown in **S5 and S6 Tables**. One hundred fifty-four antibody incubations were performed overnight at 4°C for each protein sample. For visualization, the beads were washed twice with 100 μl of PBST before species-specific PE-labeled secondary antibody (Jackson Dianova) was added in 30 μl of assay buffer for 1 hour. After two washes with 100 μl of PBST, analyte signals were generated in a FlexMAP 3D instrument (Luminex).

Data analysis: Data generated by the Luminex instrument were analyzed using a proprietary analysis tool that visualizes the fluorescent signals as bar graphs and identifies specific protein peaks detected by antibodies. Each graph was composed of the 96 values derived from the corresponding molecular mass fractions obtained after antibody incubation. The software tool identified specific peaks, and a relative molecular mass was assigned to each of the 96 fractions. After background correction, specific signal intensities were calculated as the integral of the identified peak.

### *In vivo* drug response assays and FACS analysis

Cell cultures CC0514-R1 and CC0514-R4 were transduced with lentiviruses carrying a phosphoglycerate kinase (PGK) promotor controlled eGFP (pLenti PGK GFP Puro, w509-5, Addgene) or mCherry. The pLenti PGK mCherry Puro vector construct was created via PCR cloning of mCherry cDNA from plasmid vector 7TCF (Addgene, Plasmid #24307) and modified with BamHI and BsrgI restriction sites for directed ligation into plasmid vector pLenti PGFK GFP Puro. Plasmids were tested for correct insert orientation via Sanger sequencing. Transduced cells were selected with puromycin. Following organoid cell culture expansion, cells were harvested at passage 08, digested, and filtered, and single cells were counted. Transplantation into nude mice was achieved by subcutaneous injection of single populations or 1:1 mixtures of 1.0×10^6^ cells each at EPO GmbH (Experimental Pharmacology and Oncology, Berlin-Buch, Germany). Mice (BOMTac: NMRI-Foxn1^nu^; Genotype NMRI NU-F Sp/Sp) were obtained commercially from Taconic (Lille Skensved, DK).

Organoid models were injected at 1:1 dilution with Matrigel (Corning, NL) and were allowed to expand for 10 days, reaching PDX sizes of 0.1 cm^3^, before treatment with selected compounds for 36 days. The compounds were administered either orally or intraperitoneally. Following the first day of treatment, the tumor volume and body weight were recorded twice weekly. Animal welfare was controlled regularly. Animals in poor health were sacrificed independent of treatment duration. Tumor volume was calculated from the measurement of the length and width of subcutaneous tumors (TV = (length+width)^2^/2). For further information on the handling of PDX models, please refer to [76]. Mice were sacrificed after the PDX tumors reached a size of approximately 1.5 cm^3^. Retrieved PDX tumors were subjected to whole-mount (immunofluorescence) imaging using a Zeiss Axiovert 400 microscope (Zeiss, Germany). PDX tumor tissues were digested to produce single-cell suspensions and analyzed by FACS at the German Rheumatism Research Centre (DRFZ, Berlin, Germany) to record the distribution of GFP- and mCherry-positive cells.

## Supporting information

S1 Fig Culture establishment statistics and UICC stages of donor tumors. Bar graph shows culture establishment percentages per UICC stage.
(TIF)

S2 Fig Bead-based western blot analysis of (phospho-) proteins in 9 PD3D cultures reveals sample-specific protein expression levels. Bead-based western blotting (“DigiWest”) analysis of 104 (phospho-) proteins in 9 organoid cultures and tissues (healthy and tumor). The hierarchical cluster analysis shows scaled, Strep-normalized protein expression values, color-coded from lowest (yellow) to highest (blue) expression value.
(TIF)

S3 Fig DigiWest protein expression profiles of organoids OT151, OT276 and OT347-M01 treated with gefitinib, afatinib and sapitinib. Protein expression profiling of cultured organoids derived from three different patients was performed using DigiWest and expression of 135 proteins was measured using specific antibodies. Measurements were performed on organoids that were treated for 72h with the RTK inhibitors gefitinib, afatinib and sapitinib and with vehicle control (DMSO). To visualize the effect of the different substances the ratio of the specific RTK inhibitor over the vehicle control was calculated, log2 transformed and the data was subjected to hierarchical cluster analysis (Pearson Correlation, complete linkage).
(TIF)

S4 Fig *In vivo* drug response assay for mixed subpopulations of sibling cultures CC0514-R1- and -R4. *In vivo* drug treatment of 1:1 mixed cells (1.0×10^6^ overall) of cultures CC0514-R1-GFP and CC0514-R4-mCh started 10 days post injection. Drug treatment was stopped at day 45. Line plot shows growth curves of triplicates of the respective single or combinatorial treatments. Color code is given in the legend.
(TIF)

S5 Fig Sanger sequencing of SMAD4 codon 361 in patient CC0514. Electropherograms of SMAD4 codon 361 affected by SMAD4 mutations in cultures CC0514-R1 and -R2 in comparison to SMAD4 wild-type culture CC0514-R5. For all cases, one early and later passage was tested. Blue areas indicate the respective codon, read from left to right.
(TIF)

S1 Table Description of study cohort. (XLSX)

S2 Table Panel sequencing results for 49 PD3D cultures and 29 matched tumor tissues. (XLSX)

S3 Table Compound c_ss_ values for *in vitro* assay. (XLSX)

S4 Table IC_50_- and E_max_ values of tested PD3D cultures. (XLSX)

S5 Table DigiWest assay results for tissues and organoid cultures. (XLSX)

S6 Table DigiWest assay results for organoids treated with gefitinib, afatinib and sapitinib. (XLSX)

S7 Table Consensus molecular subtypes (CMS) of CC0514 sibling cultures. (XLSX)

S8 Table Somatic mutations in patient CC0514 tumor tissues and *in vitro* cultures. (XLSX)

S9 Table Differentially expressed genes of CC0514 sibling cultures grouped by SMAD4 mutation status (R1, R2 vs. R3, R4, R5). (XLSX)

S10 Table PDX tumor volumes of single and mixed populations of CC0514-R1 and -R4 cells. (XLSX)

S11 Table PDX tumor volumes of treated mixed populations of CC0514-R1-GFP and CC0514-R4-mCh cells. (XLSX)

S12 Table FACS results—GFP^+^/mCherry^+^ fractions and fold enrichment. (XLSX)

S13 Table Panel sequencing results compared to whole exome/genome data from [11]. (XLSX)
