# Correction: Heterogeneous pathway activation and drug response modelled in colorectal-tumor-derived 3D cultures

**DOI:** 10.1371/journal.pgen.1008183

**Published:** 2019-05-29

**Authors:** The PLOS Genetics Staff

An error was introduced during the production process. The captions for Figs 2, 5, 6 and 7 are incorrectly formatted due to a typesetting error. Please see the correct figure captions here. The publisher apologizes for the error.

**Fig 2:**
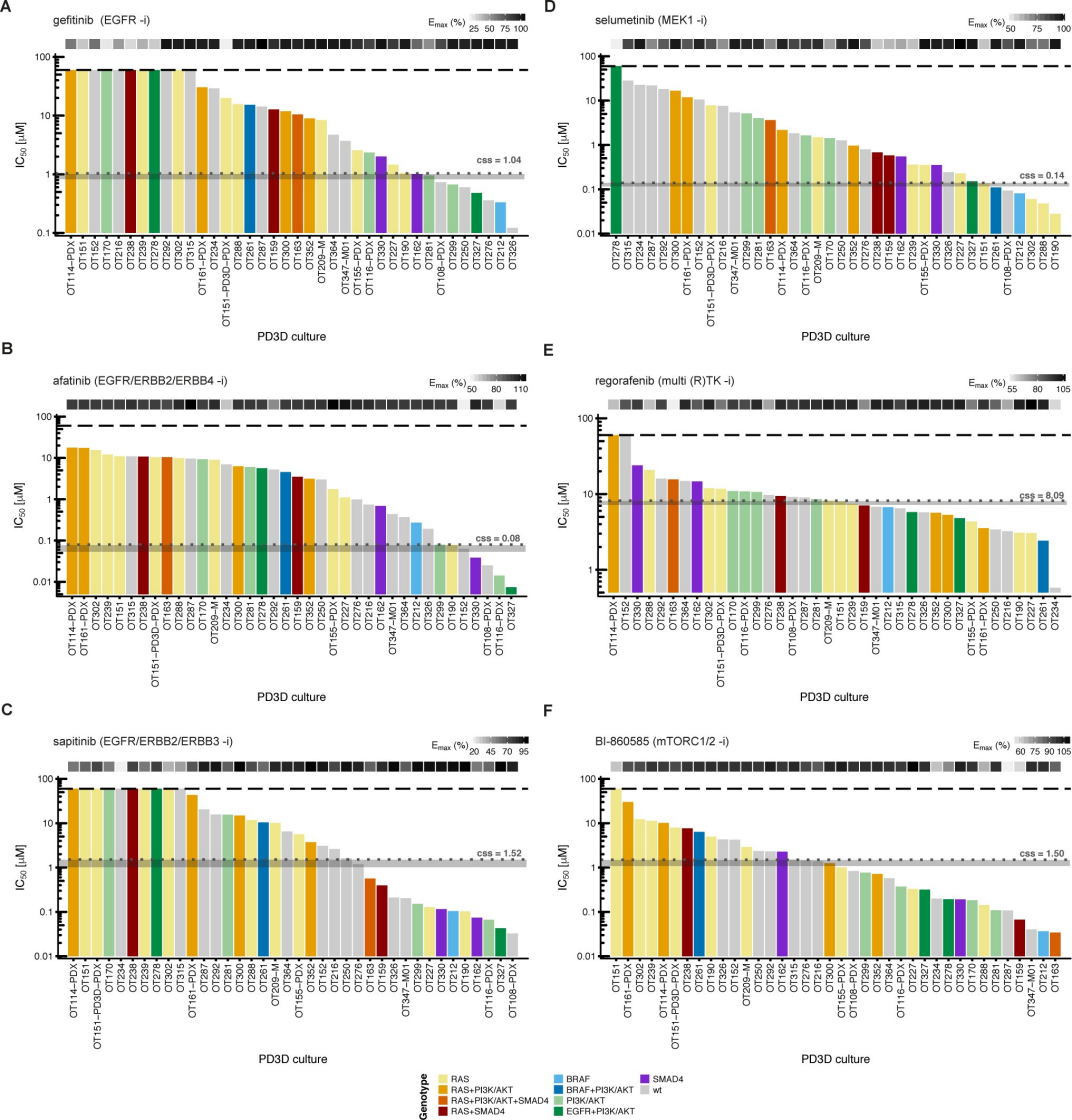
*In vitro* drug response waterfall plots of 38 organoid cultures show individual patterns of resistance and sensitivity. IC_50_ values for 38 *in vitro* models were determined with a semi-automated drug response assay platform. The lower and upper assay cutoffs were 0.003 μM and 60 μM. Drug efficacy (E_max_) was included as additional measure of response, indicated with light grey to black boxes according to percent efficacy above the waterfall plot. The genotype of each culture according to panel sequencing is color-coded according to the legend given at the bottom of the figure. Area (grey) of achievable steady state *in vivo* plasma concentrations (c_ss_) are given in μM and indicated with grey dotted lines. (A-C) IC_50_ values for small molecules gefitinib, afatinib and sapitinib, targeting the ERBB receptor(s) ERBB1/EGFR, ERBB2/Her2, ERBB3 and ERBB4. (D) Inhibition at the level of MEK1/2 with selumetinib. (E) Response to the multikinase inhibitor regorafenib. (F) Treatment with the mTORC1/2 inhibitor BI-860585. The *BRAF*^G466R^ and *BRAF*^G466V^ mutations in tumors OT170 and OT327, respectively, are not included in the figure, as their gene products are considered kinase-dead [77].

**Fig 5:**
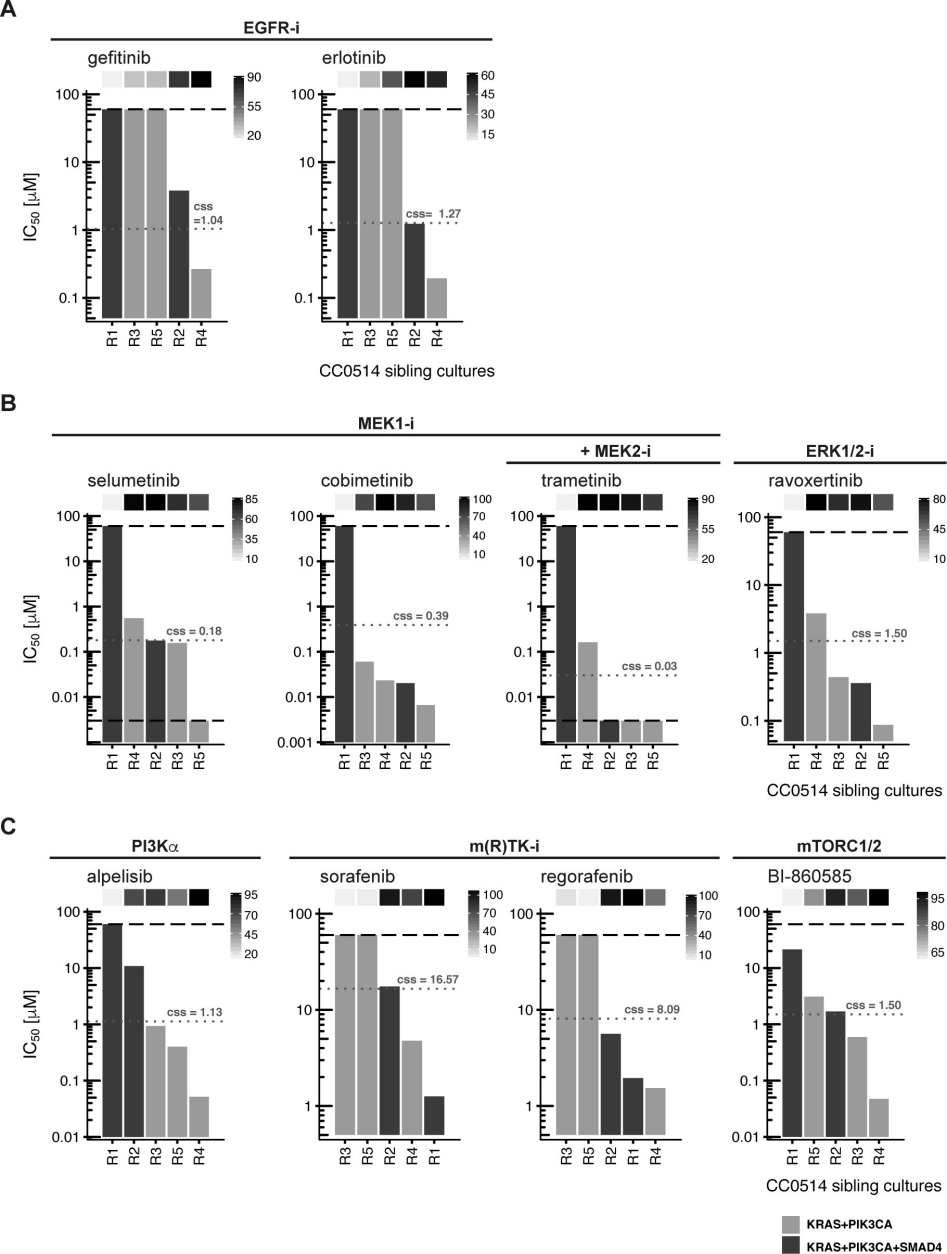
Organoid sibling cultures display heterogeneous drug responses. IC_50_ values for sibling cultures derived from patient tumor CC0514 treated with a panel of small molecules targeting EGFR, MEK/ERK, PI3Kα, mTORC1/2 and multiple kinases are shown. The lower and upper assay cutoffs were 0.003 μM and 60μM. Drug efficacy (E_max_) was included as additional parameter of response, indicated with light grey to black boxes above the waterfall plot according to percent efficacy. Critical mutations in each culture are depicted in light and dark grey bars according to the legend at the bottom of the figure. Achievable steady state *in vivo* plasma concentrations (c_ss_) are given in μM and indicated with grey dotted lines. (A) IC_50_ values found following inhibition at the EGF receptor. (B) IC_50_values found following inhibition at downstream pathway components MEK and ERK. (C) Inhibition with alpelisib (targeting PI3Kα), BI–860585 (mTORC1/2) and the multi-kinase inhibitors sorafenib and regorafenib.

**Fig 6:**
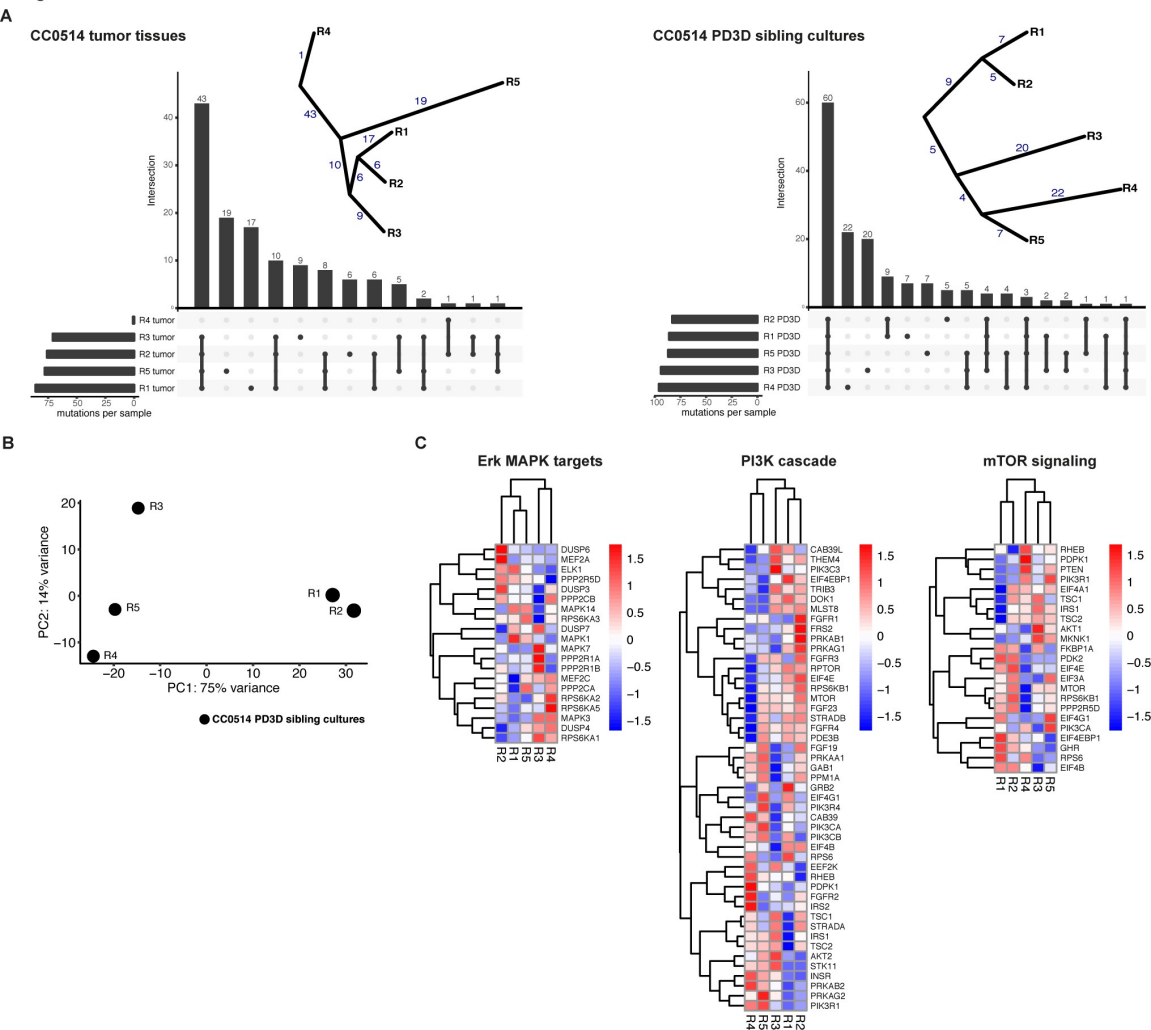
Tumor tissues and sibling cultures of patient CC0514 display genetic heterogeneity and heterogeneous mRNA expression profiles. Heterogeneity of tumor tissues and PD3D sibling cultures was evaluated on DNA and mRNA levels. (A) On the genomic level, somatic mutations were called from DNA of the five original tumor pieces (R1-R5) of the primary tumor of patient CC0514 and the respective PD3D sibling cultures, compared to CC0514 patient´s blood. Cellular content of tumor tissue R4 was very low. UpSet plots show rare somatic mutations (MAF < 0.001) in exonic regions used to construct evolutionary trees of the somatic mutations, displayed next to the plots. The numbers of shared or private mutations are given. (B) Principal Component Analysis of the mRNA expression of the sibling cultures. First component on x-axis contains 75% of the variance and classifies the samples into two major groups R1/R2 vs. R3/R4/R5). (C) Heatmaps of mRNA expression of components of ERK/MAPK, PI3K and mTOR signaling pathways. Each row has been transformed using Z-score. The color code represents the scaled mRNA expression across samples. Genes and samples were hierarchically clustered using Euclidean distance.

**Fig 7:**
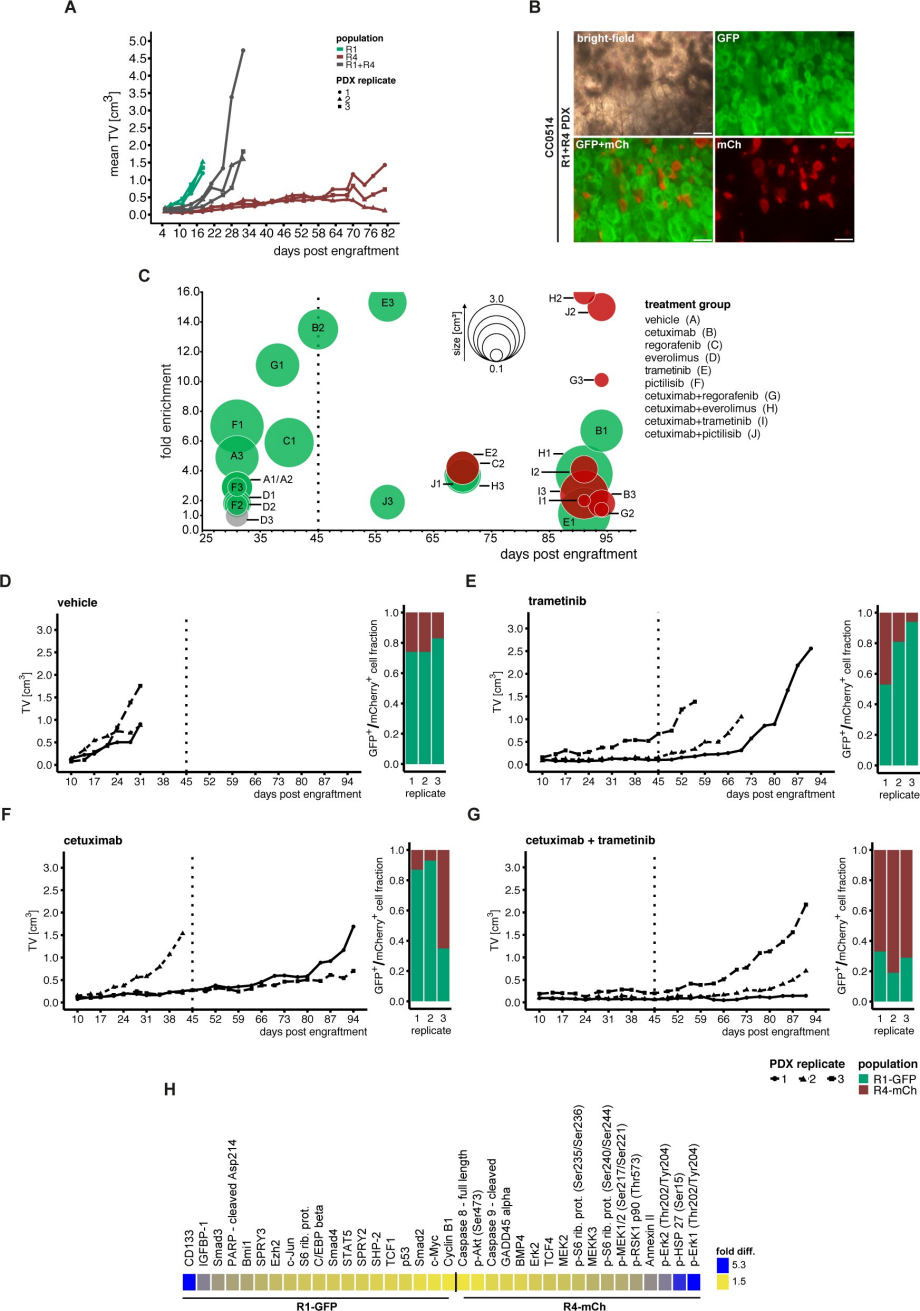
Subpopulation-specific response to *in vivo* drug treatment. (A) Cells of sibling cultures CC0514-R1 and CC0514-R4 were transduced with PKF-GFP and PGK-mCherry (mCh) markers, respectively. 1.0×10^6^ cells were injected into nude mice either as single populations (green/red) or as a 1:1 mixture of both populations (grey) in triplicate. (B) Microscopic images of a mixed R1-PGK-GFP and R4-PGK-mCherry tumor, scale bars: 200μm. (C) Mixed populations of R1 and R4 cultures were subjected to treatment *in vivo* in triplicate. Treatments started 10 days post injection included 5 single compounds and combinations with cetuximab. Treatments were carried out until 45 days (dashed line), if possible. PDX tumors showing minor growth post treatment were maintained *in vivo* to monitor subsequent growth. The bubble plot shows tumor sizes, as represented by bubble diameter, and fold enrichment of cell subpopulations for all replicates in treatment groups A-J (displayed in the figure). Green (= GFP^+^) or red (mCherry^+^ = mCh^+^) fills indicate which subpopulation was more abundant in the PDX tumor, as measured by FACS analysis of re-suspended tumor cells. Grey circles indicate a 50:50 distribution of labelled tumors. Note that for PDX tumors C2 and E2 both fold enrichment and tumor size were at a similar range (S11 and S12 Tables). (D-G) Tumor growth kinetics during the course of the *in vivo* mixing experiment are shown together with the fractions of GFP^+^ and mCh^+^ populations at the end of the experiment (FACS analysis). Treatments were done with vehicle, trametinib, cetuximab and cetuximab+trametinib combination. (H) Protein expression of CC0514-R1-GFP and CC0514-R4-mCh organoids analyzed by DigiWest protein assay. Difference in fold expression ranging from 1.5 (yellow) to 5.3 (blue).
