# Supplementary figures and images for: Heterogeneous pathway activation and drug response modelled in colorectal-tumor-derived 3D cultures
Source: PLoS Genet. 2019 Mar 29;15(3):e1008076. doi: 10.1371/journal.pgen.1008076 (PMC6457557; doi:10.1371/journal.pgen.1008076)

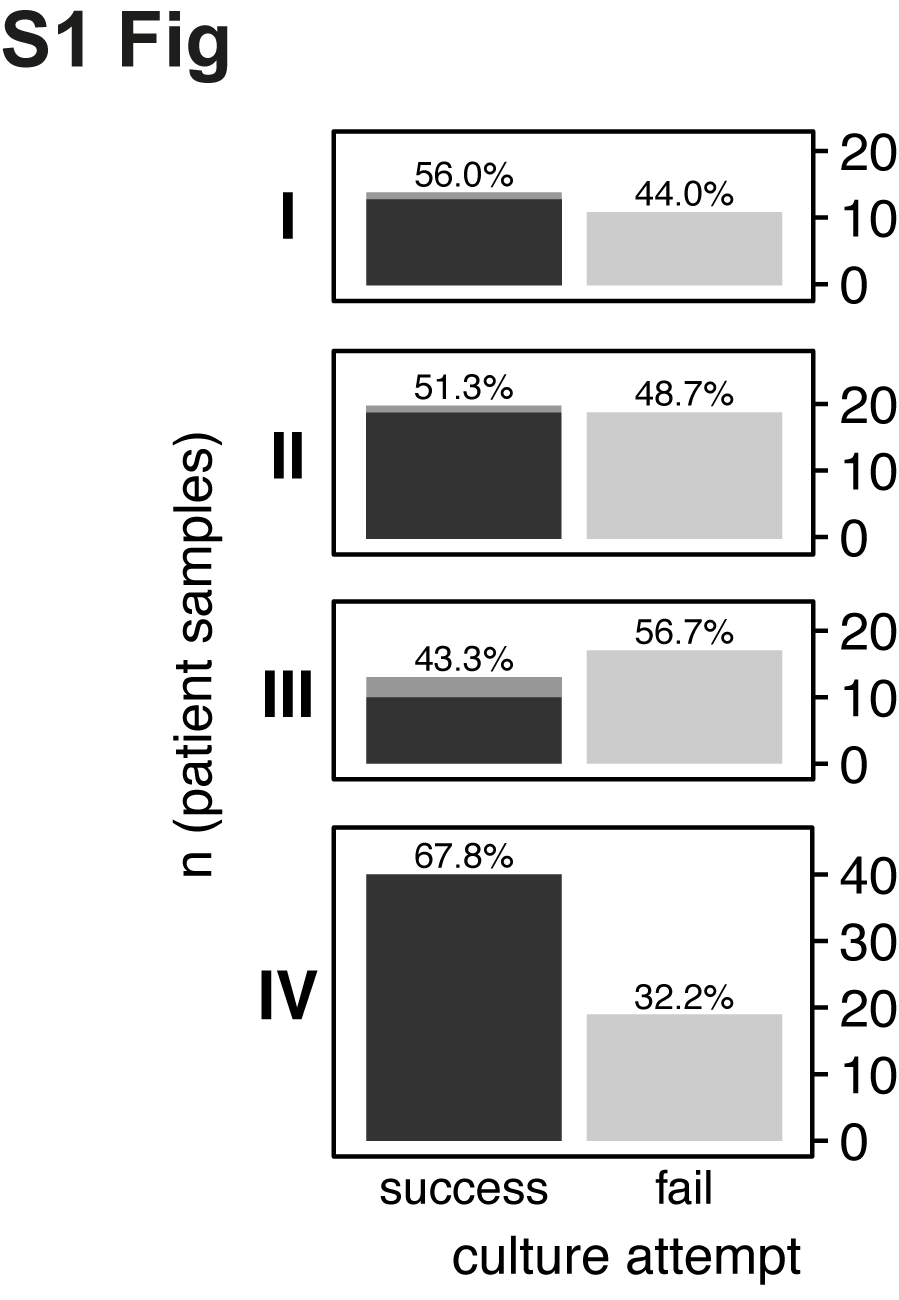

Supplement: S1 Fig — Bar graph shows culture establishment percentages per UICC stage. (TIF) [file pgen.1008076.s001.tif]

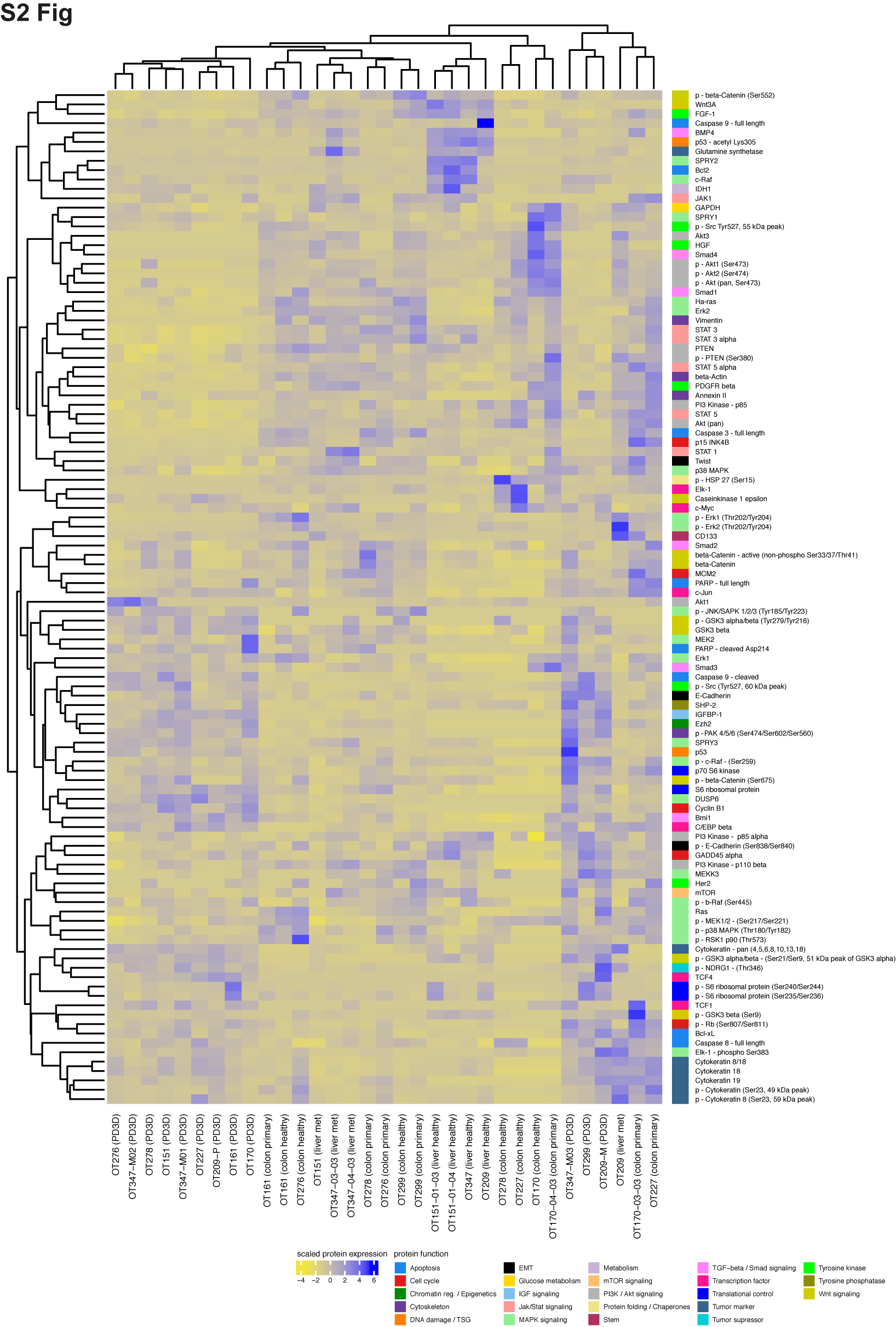

Supplement: S2 Fig — Bead-based western blotting (“DigiWest”) analysis of 104 (phospho-) proteins in 9 organoid cultures and tissues (healthy and tumor). The hierarchical cluster analysis shows scaled, Strep-normalized protein expression values, color-coded from lowest (yellow) to highest (blue) expression value. (TIF) [file pgen.1008076.s002.tif]

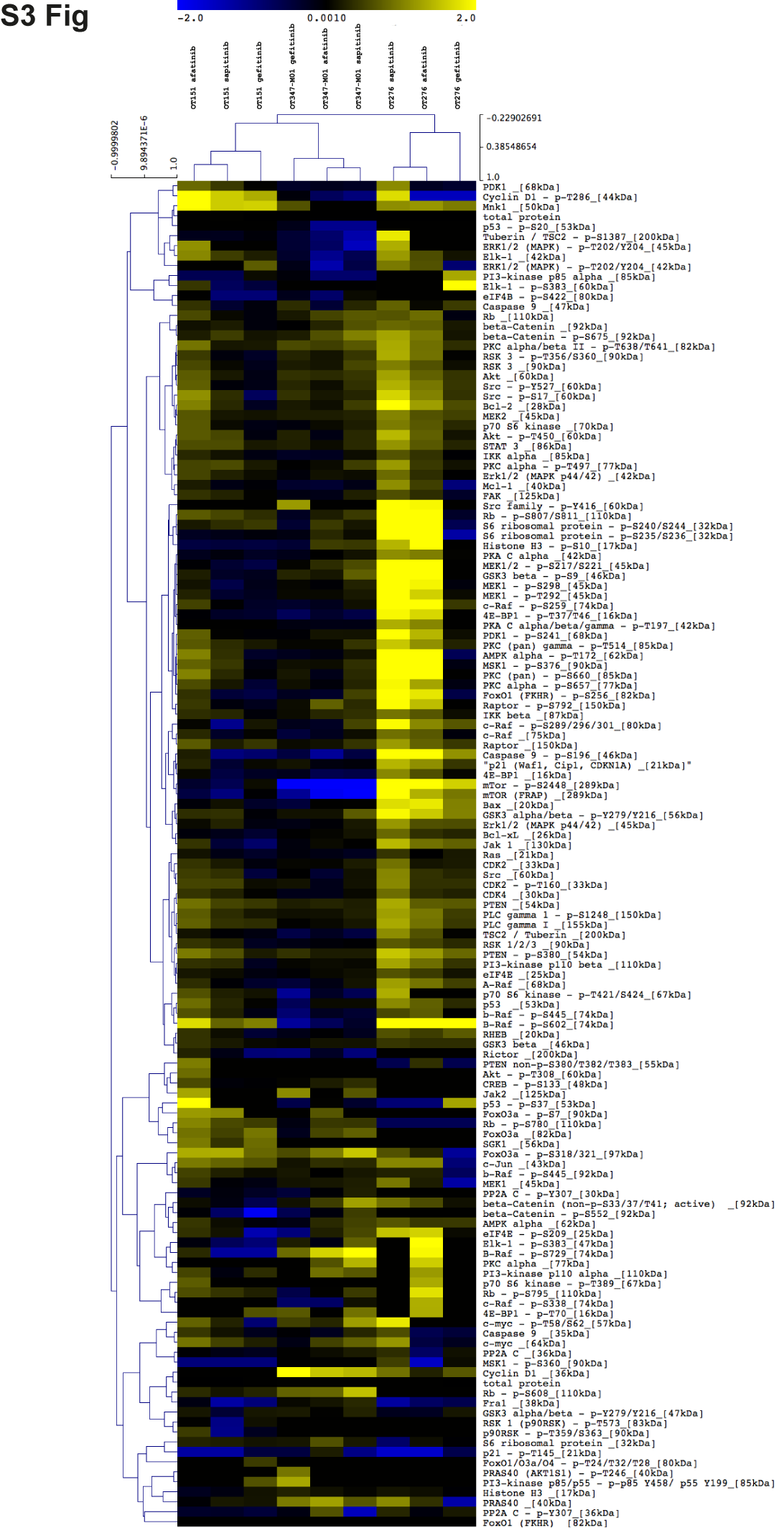

Supplement: S3 Fig — Protein expression profiling of cultured organoids derived from three different patients was performed using DigiWest and expression of 135 proteins was measured using specific antibodies. Measurements were performed on organoids that were treated for 72h with the RTK inhibitors gefitinib, afatinib and sapitinib and with vehicle control (DMSO). To visualize the effect of the different substances the ratio of the specific RTK inhibitor over the vehicle control was calculated, log2 transformed and the data was subjected to hierarchical cluster analysis (Pearson Correlation, complete linkage). (TIF) [file pgen.1008076.s003.tif]

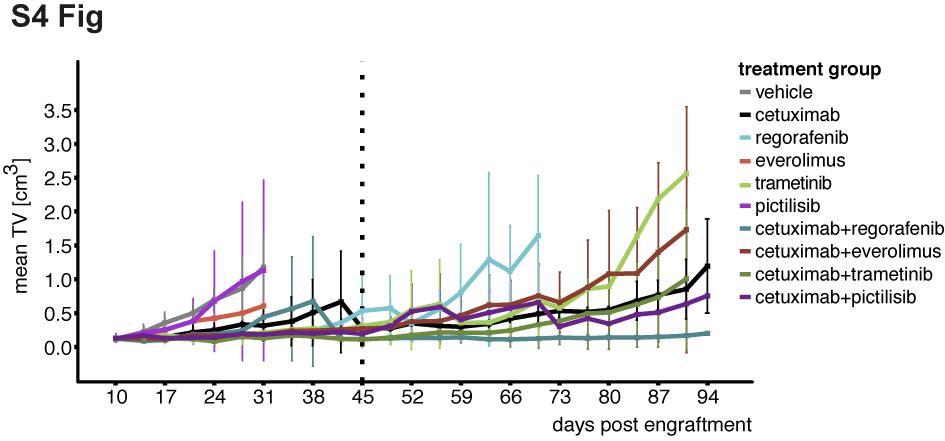

Supplement: S4 Fig — In vivo drug treatment of 1:1 mixed cells (1.0×10^6^ overall) of cultures CC0514-R1-GFP and CC0514-R4-mCh started 10 days post injection. Drug treatment was stopped at day 45. Line plot shows growth curves of triplicates of the respective single or combinatorial treatments. Color code is given in the legend. (TIF) [file pgen.1008076.s004.tif]

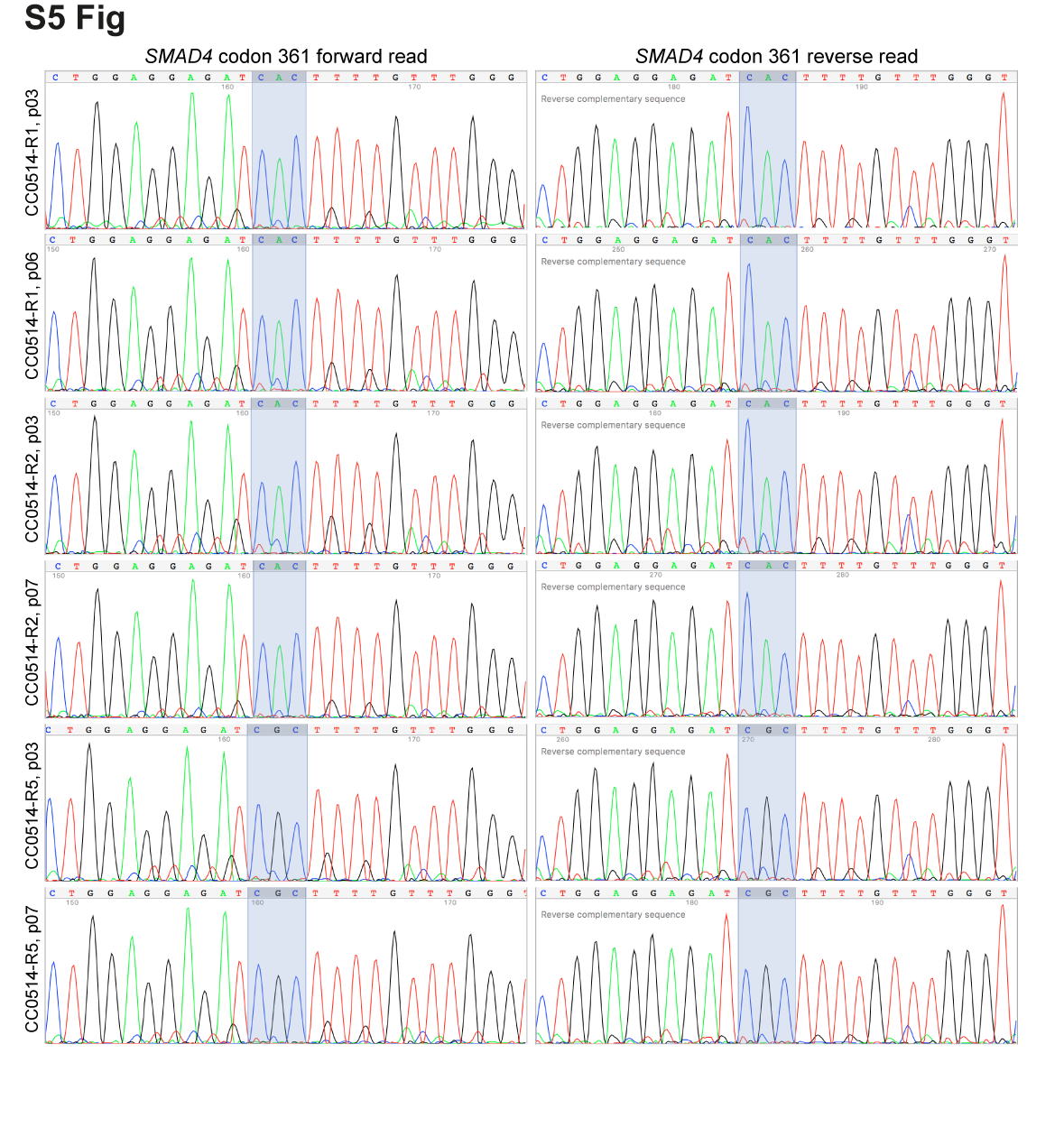

Supplement: S5 Fig — Electropherograms of SMAD4 codon 361 affected by SMAD4 mutations in cultures CC0514-R1 and -R2 in comparison to SMAD4 wild-type culture CC0514-R5. For all cases, one early and later passage was tested. Blue areas indicate the respective codon, read from left to right. (TIF) [file pgen.1008076.s005.tif]
